# Cultivar-specific gene modulation in *Vitis vinifera*: analysis of the promoters regulating the expression of WOX transcription factors

**DOI:** 10.1038/srep45670

**Published:** 2017-03-30

**Authors:** Paolo Boccacci, Anita Mela, Catalina Pavez Mina, Walter Chitarra, Irene Perrone, Ivana Gribaudo, Giorgio Gambino

**Affiliations:** 1 Institute for Sustainable Plant Protection, National Research Council (IPSP-CNR), Torino. Strada delle Cacce 73, 10135 Torino, Italy; 2 Institute for Sustainable Plant Protection, National Research Council (IPSP-CNR), Grugliasco Unit. Largo P. Braccini 2, 10095 Grugliasco-TO, Italy

## Abstract

The family of *Wuschel-related Homeobox* (*WOX*) genes is a class of transcription factors involved in the early stages of embryogenesis and organ development in plants. Some of these genes have shown different transcription levels in embryogenic tissues and mature organs in two different cultivars of *Vitis vinifera*: ‘Chardonnay’ (CH) and ‘Cabernet Sauvignon’ (CS). Therefore, we investigated the genetic basis responsible for these differences by cloning and sequencing in both the cultivars the promoter regions (~2000 bp) proximal to the transcription start site of five *VvWOX* genes. We then introduced these promoters into *Arabidopsis thaliana* for expression pattern characterisation using the *GUS* reporter gene. In the transgenic *Arabidopsis*, two promoters isolated from CS (*pVvWOX13C_CS* and *pVvWOX6_CS*) induced increased expression compared to the sequence isolated in CH, confirming the data obtained in grapevine tissues. These results were corroborated by transient expression assays using the agroinfiltration approach in grapevine somatic embryos. Truncated versions of *pVvWOX13C* demonstrated that few nucleotide differences between the sequences isolated from CH and CS are pivotal for the transcriptional regulation of *VvWOX13C*. Analysis of promoters using heterologous and homologous systems appear to be effective for exploring gene modulation linked with intervarietal sequence variation in grapevine.

The regulation of gene transcription in eukaryotes is a complex mechanism that requires precise interactions among several proteins and DNA sequences. Promoters are cis-regulatory elements composed of non-coding DNA containing binding sites for transcription factors (TFs), which activate and sustain the transcription of genes^1^. The spatial and temporal regulation of the gene transcription is an important system influencing the plant development and the response to environmental stimuli such as light, biotic and abiotic stresses. Furthermore, the mutations affecting the gene regulation were considered important sources of evolutionary change and could be one of the most important cause of morphological divergence between organisms^1^,^2^. Indeed, the mutations in protein-coding regions could have more pleiotropic effects, because generally they affect all tissues in which the protein is active, whereas the change of a tissue-specific regulatory element in the promoter should affect only the cells interested by the specific expression change^1^,^2^. In this context, the study of promoters becomes particularly important for deepening the genetic basis of phenotypic variants in species such as grapevine (*Vitis vinifera* L.), characterized by thousands of different cultivars highly heterozygous^3^. In the last years, there has been a new impetus to study promoters in grapevine^4^,^5^, which arises from the applications of new “Sustainable Biotechnology”, i.e. cis-genesis and genome editing^6^,^7^, for which it is very important to know and access many regulatory sequences.

The *WUSCHEL (WUS)-related Homeobox (WOX)* gene family is a class of homeodomain transcription factors involved in plant development by regulation of cell division and differentiation^8^. These genes were first characterised in *Arabidopsis*^8^, and in the following years their function has been analysed in several species^9^,^10^,^11^,^12^. The *WOX* family consists of 15 members in the *Arabidopsis* genome^8^. The first identified *WUS* is required for the maintenance of stem cells in the shoot apical meristem^13^. The other members are involved in many different phases of plant development: *WOX5* performs a function similar to that of *WUS* in the root meristem^14^,^15^ and *WOX4* regulates the vascular cellular division^16^. *WOX6* is required for ovule development^17^, and *WOX2, WOX8* and *WOX9* are important cell fate regulators of early pre-embryos^8^,^18^. In addition, these transcription factors generally show a high functional redundancy^19^, and some remain partially uncharacterised due to a lack of clear phenotypes in loss-of-function mutants.

In grapevine, 12 *VvWOX* genes were previously identified and their expression levels were analysed in somatic embryogenic tissues^20^. Somatic embryogenesis is the regenerative process most used in grapevine for genetic transformation^21^. However, it is negatively affected by many factors, such as genotype: indeed cultivars of *V. vinifera* vary widely in their potential in forming embryogenic tissues, and some are particularly recalcitrant^22^. *VvWOX* genes are important regulators of somatic embryogenesis in grapevine^20^ and, interestingly, their regulation during the early phase of the regenerative process differs between the two cultivars ‘Chardonnay’ (CH) and ‘Cabernet Sauvignon’ (CS), showing respectively high and low aptitude to embryogenesis. *VvWOX9* is the main *WOX* gene expressed during the somatic embryogenesis process, and the low aptitude for embryogenesis observed in CS could be partially correlated with low expression levels of this gene^20^. In addition, other *VvWOX* genes have showed significant expression differences between CH and CS in different tissues. In particular, *VvWOX3, VvWOX4, VvWOX6, VvWOX13A* and *VvWOX13C* are expressed at high levels in undifferentiated calli of CS; *VvWOX1* and *VvWOX2* are expressed at high levels in embryogenic calli of CH; and *VvWOX6* is expressed at a high level in the anthers of CS^20^.

In this work, five *VvWOX* genes showing the higher expression differences between CH and CS and belonging to the different evolutionary lineages previously reported: modern (*VvWOX1, VvWOX4* and *VvWOX6*) intermediate (*VvWOX9*) and ancient clade (*VvWOX13C*)^20^ were chosen for further analyses. The transcriptional characterisation of these genes was extended to several grapevine organs, and promoter regions from CH and CS, proximal to transcription start sites, were cloned for the production of promoter::GUS fusion constructs. In transgenic *Arabidopsis* and in agroinfiltrated somatic embryos of grapevine, some point mutations associated with transcription factor binding sites (TFBSs) resulted responsible for a higher level of transcription induced by the promoters of *VvWOX13C* and *VvWOX6* isolated from CS (*pVvWOX13C_CS* and *pVvWOX6_CS*).

## Results and Discussion

### Expression of *VvWOX* genes in different grapevine organs

The analysis of transcript levels of five grapevine genes (*VvWOX1, VvWOX4, VvWOX6, VvWOX9* and *VvWOX13C*), showing different regulation in embryogenic tissues of CH and CS^20^, was extended to different organs at different developmental stages in plants of CH and CS cultivated in field conditions.

**Figure 1:**
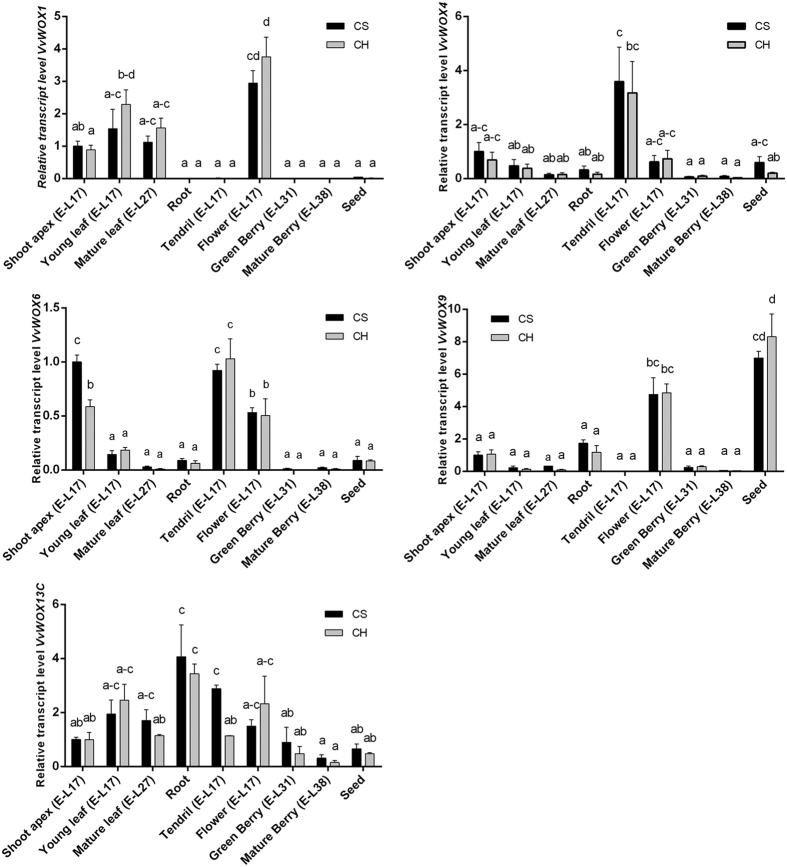
Relative expression level of *VvWOX1, VvWOX4, VvWOX6, VvWOX9* and *VvWOX13C* in different organs of ‘Chardonnay’ (CH) and ‘Cabernet Sauvignon’ (CS) as determined by qRT-PCR. Lowercase letters denote significant differences attested by Tukey’s HSD test (P < 0.05). Data are expressed as means ± SE (n = 3).

*VvWOX1* was expressed in a specific way in leaves, shoot apexes and in flowers, while no expression was detected in other organs, except for a faint signal in seeds (Fig. 1). This expression pattern confirms the previous observations in grapevine^20^ and in other plants. Indeed, in *Arabidopsis, WOX1* was initially identified in the initiating vascular primordium of the cotyledons during the heart and torpedo stages of the embryos^8^. Since then, it has been demonstrated that *WOX1,* in association with *WOX3/PRESSED FLOWER*, is expressed in a middle domain downstream of adaxial/abaxial polarity establishment in the leaf primordia and promotes leaf blade outgrowth and margin development^23^. This close association between *WOX1* and leaf morphology was also observed in *Populus tomentosa*^24^ and *Pisum sativum*^25^, in which this gene is also involved in flower development, such as in *Medicago truncatula*^26^.

The expression of *VvWOX6* was concentrated in young tissue as the shoot apexes, in flowers and tendrils, while in mature organs such as fully developed leaves and berries the expression decreased significantly (Fig. 1). *VvWOX9* was expressed at high levels in seeds and flowers, at lower levels in the root and shoot apexes, and was almost absent in other organs (Fig. 1). The expression in shoot apexes substantially reflects the activity of *WOX9* as regulator of *WUS* expression in the shoot apical meristem^27^.

*VvWOX4* was expressed in almost all grapevine organs analysed, particularly in tendrils (Fig. 1). This gene is a key regulator of auxin-dependent cambium stimulation in the main stem of *Arabidopsis*^16^,^28^ and regulates vascular cellular division^29^. The high expression of *VvWOX4* in tendrils observed in grapevine could be related to their high level of vascularisation (similar to that of stems). Finally, *VvWOX13C*, which belongs to the *WOX13* subfamily and is involved in replum development, lateral root formation, and vegetative to floral growth transition^30^,^31^, was expressed in all grapevine organs analysed (Fig. 1).

Interestingly, for all genes considered, expression in berries was absent or very limited, suggesting little involvement of these genes in the berry development. In addition, the expression of *VvWOX1, VvWOX4* and *VvWOX9* changed considerably between flowers and tendrils, different structures generated from a common primordium: *Vitis* tendrils are modified reproductive organs adapted to climb^32^. These high transcriptional differences, especially for *VvWOX1*, might suggest a direct involvement of these transcription factors in the regulation of the transition of lateral meristems to flowers or tendrils in grapevine.

The differences between CH and CS in the level of expression of *VvWOX1, VvWOX4, VvWOX6, VvWOX9* and *VvWOX13C* observed during the embryogenetic process^20^ were only partially confirmed in the grapevine organs. In CS the high expression of *VvWOX6* and *VvWOX13C* observed in shoot apexes and tendrils respectively (Fig. 1), confirmed the expression levels in CS previously reported^20^. Conversely, no significant difference between the two cultivars was observed for *VvWOX1, VvWOX4,* and *VvWOX9* (Fig. 1). The reasons for these discrepancies may be multiple. For example, some differences between the two cultivars can only be highlighted in embryogenic tissues. Moreover, in the *in vitro* culture, the presence of high concentrations of plant growth regulators (PGRs)^20^ may be responsible for different transcriptional modulation between CH and CS, which is not always detectable in plants cultivated in filed conditions.

### Sequencing and computational analyses of promoters isolated from *VvWOX* genes

In order to deepen the understanding of the molecular bases of gene modulation observed in CH and CS, the promoter sequences associated to five *VvWOX* genes were analysed. Using the grapevine genome sequence PN40024^33^, regions (~2000 bp) proximal to the transcription start site of each gene were cloned and sequenced. The sequences of promoters (*pVvWOX*) deposited in GenBank under accession numbers from KY492279 to KY492288 were aligned in order to identify nucleotide differences between the sequences isolated from CH and CS (Supplementary Fig. S1). For each gene, the percentage identity between the promoters isolated from the two cultivars ranged from 96.7% for *pVvWOX4_CH* vs *pVvWOX4_CS* to 99.8% for *pVvWOX1_CH* vs *pVvWOX1_CS*. The differences in some cases were limited to a few point mutations as in *pVvWOX1_CH* vs *pVvWOX1_CS*, while in other promoters a combination of several point mutations and small deletions (*pVvWOX6_CH* vs *pVvWOX6_CS* and *pVvWOX13C_CH* vs *pVvWOX13C_CS*) were observed. Interestingly, *pVvWOX4_CS* was characterised by a long deletion of 60 bp at the position -703 to transcription start site (Supplementary Fig. S1).

**Figure 2:**
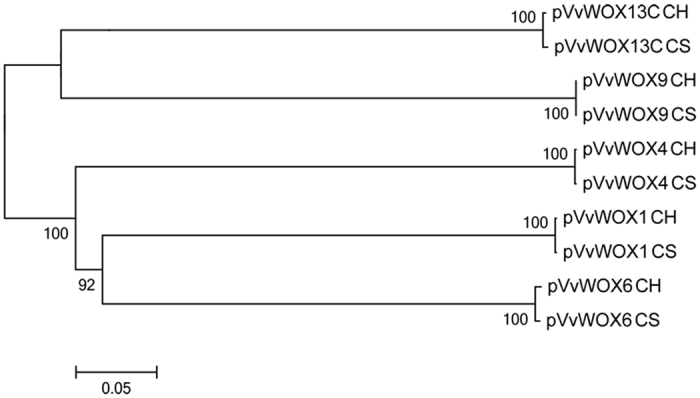
The Neighbor-Joining tree of the promoters *pVvWOX1, pVvWOX4, pVvWOX6, pVvWOX9* and *pVvWOX13C* isolated from ‘Chardonnay’ (CH) and ‘Cabernet Sauvignon’ (CS). The significance of each node was tested using 1000 bootstrap replicates.

In a phylogenetic analysis, the promoter sequences were divided in two major groups in which *pVvWOX1, pVvWOX4* and *pVvWOX6* constituted a separate clade from *pVvWOX9* and *pVvWOX13C* (Fig. 2). The relationship among these promoters is typical of a subdivision into evolutionary lineages previously reported for the WOX proteins in grapevine and other species: a modern clade (WUS, WOX1, 2, 3, 4, 5, 6, and 7) separated from an intermediate clade (WOX8, 9, 11, and 12) and an ancient clade (WOX10, 13, and 14)^8^,^20^,^34^. These results showed that the evolutionary relations among different WOX proteins can also be conserved in promoter sequences, suggesting a parallel evolution between coding sequences and gene regulation^35^.

Since even few mutations in regulatory sequences can modify the regulation of a gene^1^, the TFBSs present in the promoter sequences and their corresponding TFs were analysed by the software PlantPAN 2.0^36^. In *pVvWOX1* and *pVvWOX9* the small differences retrieved in the nucleotide sequences isolated from CH and CS (Supplementary Fig. S1) imply few changes in the potential TFBSs predicted using a bioinformatics approach (Supplementary Table S1). Conversely, in *pVvWOX4_CS,* the deletion of 60 bp (Supplementary Fig. S1) determined the loss of several TFBSs in comparison to *pVvWOX4_CH*, and point mutations reported in *pVvWOX6_CH* vs *pVvWOX6_CS* and *pVvWOX13C_CH* vs *pVvWOX13C_CS* produced several variations in the predicted TFBSs (Supplementary Fig. S2, Supplementary Table S1). Interestingly, in *pVvWOX13C_CS* a TATA-box was identified at position -393 not present in *pVvWOX13C_CH*, due to a single nucleotide mutation at position -387 (Supplementary Fig. S2, Supplementary Table S1). Several TFs linked to the regulation of development (Homeodomain, Growth-regulating factor-GRF, Squamosa promoter binding protein-SBP, Alpha-amylase, WOX), response to hormones (AUX/IAA, ARF, AP2-ERF, CAMTA, EIN3) and response to stresses (Dehydrin, LEA5, NAC, WRKY) were identified (Supplementary Table S2). In addition, *pVvWOX4, pVvWOX9* and *pVvWOX13C* contained some TFBSs putatively recognised by WOX TFs (Supplementary Table S2), suggesting a strong interaction between the components of this gene family and a possible self-regulation of these TFs. A high number of sequences recognised by B3 domain-containing TFs, involved in embryo and flower developments^37^, and sequences recognised by TFIID (TATA-box binding protein), which contribute to the expression of most RNA polymerase II-transcribed genes^38^, was present in *pVvWOX6_CS* and *pVvWOX13C_CS* (Supplementary Table S2).

Overall analyses suggested that the promoters of *VvWOX4, VvWOX6* and *VvWOX13C* showed more variations between CH and CS in comparison to the promoters of *VvWOX1* and *VvWOX9*. Therefore, subsequent analyses have been limited to *pVvWOX4, pVvWOX6* and *pVvWOX13C*.

### Characterisation of promoters isolated from *VvWOX* genes in *Arabidopsis thaliana*

The sequences of *pVvWOX4, pVvWOX6* and *pVvWOX13C* isolated from CH and CS were cloned in the expression vector pMDC164^39^ for the production of promoter::GUS fusion constructs (Supplementary Fig. S3). The constructs were inserted in *Arabidopsis* and transcriptional and histochemical analyses in different organs were carried out. *Cauliflower mosaic virus* 35S promoter inserted in pMDC164 (*p35S::GUS*) and the empty vector (*p0::GUS*) were used for *Arabidopsis* transformation as positive and negative controls, respectively, (Supplementary Fig. S4).

**Figure 3:**
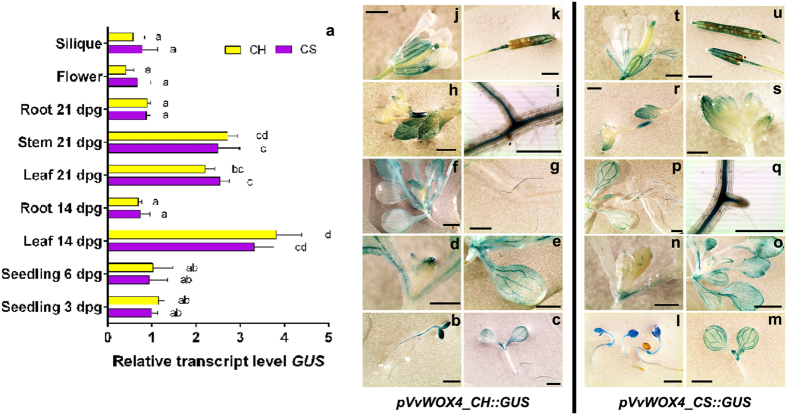
Characterisation of *pVvWOX4* isolated from ‘Chardonnay’ (CH) and ‘Cabernet Sauvignon’ (CS) in *Arabidopsis thaliana*. (**a**) Relative expression level of *GUS* under control of *pVvWOX4_CH* and *pVvWOX4_CS* in transgenic *Arabidopsis* organs collected at different developmental stages. Lowercase letters denote significant differences attested by Tukey’s HSD test (P < 0.05). Data are expressed as means ± SE (n = 3). (**b**–**u**) Histochemical assay for GUS expression in transgenic *Arabidopsis* organs. Data shown are representative of three independent transgenic lines. Size bar = 2 mm.

The histochemical assay for determining GUS activity in *Arabidopsis* plantlets at 3 days post germination (3 dpg) showed a very intense blue staining induced by both the constructs *pVvWOX4_CH::GUS* and *pVvWOX4_CS::GUS* in cotyledons and in all the other organs including roots (Fig. 3b,c,l). At 6 dpg the GUS activity started during leaf development at the apex (Fig. 3d,n,m), and increased to higher levels in adult leaves, where intense blue staining was observed in veins at 14 and 21 dpg (Fig. 3e,f,o). During development, the primary roots showed a discontinuous blue staining at 14 and 21 dpg, with an irregular activity of GUS in old tissues, while expression remained high in younger secondary roots (Fig. 3g,i,p,q). The young flower organs appeared less coloured, as reported for the young leaves, while during flower development the sepals, the filaments of anthers, and the stigma showed a high GUS activity (Fig. 3h,j,r,s,t). The siliques appeared intensely stained and no blue signal was detected in the external teguments of the seeds (Fig. 3k,u). The quantification by qRT-PCR of *GUS* expression confirmed that the mature leaves at 14 and 21 dpg, and the inflorescence stems are the organs with highest expression (Fig. 3a). Interestingly, the distribution of GUS activity in *Arabidopsis* organs driven by *pVvWOX4* appears correlated to the biological functions of *WOX4,* linked to cambium differentiation in the stem^16^,^28^, suggesting that *Arabidopsis* is an adequate system for the characterisation of promoters of *VvWOX* genes.

There was no significant difference between the expression levels of the constructs *pVvWOX4_CH::GUS* and *pVvWOX4_CS::GUS* in all organs and in all developing stages of transgenic plants (Fig. 3). Consequently, the deletion of 60 bp in the *pVvWOX4_CS* and the differences in the putative TFBSs (Supplementary Fig. S2) do not seem to influence the transcription of *GUS* in *Arabidopsis*.

In order to verify whether *pVvWOX4_CH::GUS* and *pVvWOX4_CS::GUS* are influenced by PGRs, plantlets at 6 dpg were transferred to a medium containing 2,4-dichlorophenoxyacetic acid (2,4-D) and 6-Benzyladenine (BA) at the same concentrations used to induce embryogenesis in grapevine^20^. After 8 days of culture, *GUS* expression was stable in leaves independently of conditions (with or without PGRs) and constructs, while in roots cultured on PGRs the expression increased significantly for both the constructs (Supplementary Fig. S5a). Interestingly, the *pVvWOX4* contains some regulatory regions influenced by PGRs, while even in presence of PGRs no expression difference between *pVvWOX4_CH::GUS* and *pVvWOX4_CS::GUS* was observed.

**Figure 4:**
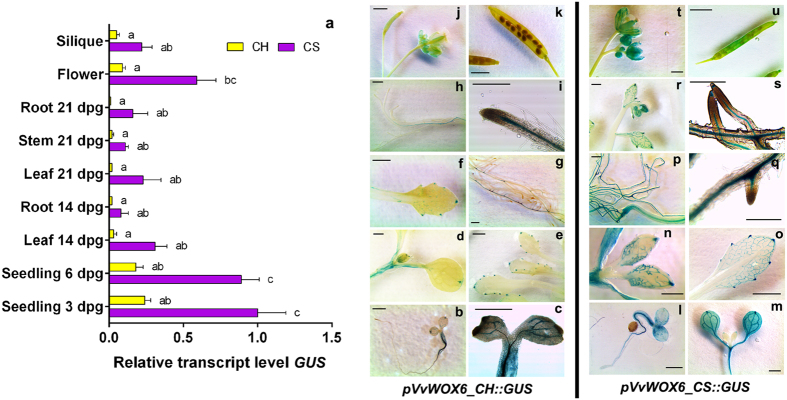
Characterisation of *pVvWOX6* isolated from ‘Chardonnay’ (CH) and ‘Cabernet Sauvignon’ (CS) in *Arabidopsis thaliana*. (**a**) Relative expression level of *GUS* under control of *pVvWOX6_CH* and *pVvWOX6_CS* in transgenic *Arabidopsis* organs collected at different developmental stages. Lowercase letters denote significant differences attested by Tukey’s HSD test (P < 0.05). Data are expressed as means ± SE (n = 3). (**b**–**u**) Histochemical assay for GUS expression in transgenic *Arabidopsis* organs. Data shown are representative of three independent transgenic lines. Size bar = 2 mm.

In *Arabidopsis* plantlets at 3 dpg, several differences in GUS activity between *pVvWOX6_CH::GUS* and *pVvWOX6_CS::GUS* were detected (Fig. 4). Seedlings transformed with *pVvWOX6_CH::GUS* showed an intense staining in hypocotyl and root, decreasing toward the root tip. In cotyledons blue signals were limited to the veins and to some cells at the end of the cotyledon (Fig. 4b,c). In contrast, seedlings under the control of the promoter isolated from CS showed an intense blue staining in all parts of the plantlets, particularly on the cotyledons, in the hypocotyl and in the vascular system along the root (Fig. 4l). In both constructs, the activity of GUS at 6 dpg was stable in cotyledons and roots, while the young emerging leaves were less stained, with some signal localised in the veins and at the apical extremity (Fig. 4d,m,n). At 14 and 21 dpg in the plants transformed with *pVvWOX6_CH::GUS,* the blue staining was limited to a few groups of cells on the edges of leaves and in roots, which gradually lost the GUS activity in the older tissues (Fig. 4e–i). Conversely, plants transformed with *pVvWOX6_CS::GUS* at 14 and 21 dpg, showed a general more intense blue staining. In addition, in leaves the midribs were not stained, but the secondary veins appeared blue (Fig. 4o–s). In flowers, sepals and styles were intensely stained in *pVvWOX6_CS::GUS,* with activity also detectable in the siliques (Fig. 4t,u). In *pVvWOX6_CH::GUS* transgenic plants, the activity was lower in flowers (Fig. 4j) and not detectable in siliques (Fig. 4k). The qRT-PCR analyses confirmed a higher expression associated with *pVvWOX6_CS::GUS*; in all organs, the promoter isolated from CS induced a *GUS* expression at least five times higher than that induced by *pVvWOX6_CH* (Fig. 4a). These results suggest that the mutations identified in these promoters (Supplementary Figs S1 and S2) are involved in the different transcriptional regulation of *VvWOX6* in the two grapevine cultivars^20^ (Fig. 1). In particular, the mutations around the position -478, associated with changes in several TFBSs, could be potentially good candidates to explain this different gene regulation (Supplementary Fig. S2, Supplementary Table S1). In addition, as observed for *pVvWOX4*, the promoters of *VvWOX6* in transgenic *Arabidopsis* induced a *GUS* expression in agreement with the expression reported in grapevine. In particular, the expression of both constructs in *Arabidopsis* was localised in young organs, seedlings at 3 and 6 dpg, and in flowers (Fig. 4A), as well as high expression was detected in shoot apexes and in flowers of grapevine (Fig. 1).

**Figure 5:**
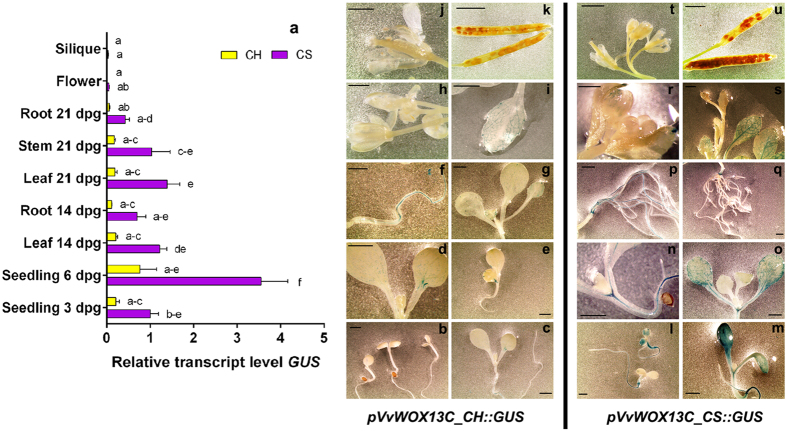
Characterisation of *pVvWOX13C* isolated from ‘Chardonnay’ (CH) and ‘Cabernet Sauvignon’ (CS) in *Arabidopsis thaliana*. (**a**) Relative expression level of *GUS* under control of *pVvWOX13C_CH* and *pVvWOX13C_CS* in transgenic *Arabidopsis* organs collected at different developmental stages. Lowercase letters denote significant differences attested by Tukey’s HSD test (P < 0.05). Data are expressed as means ± SE (n = 3). (**b**–**u**) Histochemical assay for GUS expression in transgenic *Arabidopsis* organs. Data shown are representative of three independent transgenic lines. Size bar = 2 mm.

In Fig. 5 is reported the evolution of the expression of the *GUS* gene under the control of the promoters *pVvWOX13C_CH* and *pVvWOX13C_CS* in *Arabidopsis*. In seedlings at 3 dpg the first differences between the two constructs were observed; the promoter isolated from CS induced GUS activity in all parts of the plantlets, particularly in hypocotyls and roots (Fig. 5l), while the activity of *pVvWOX13C_CH::GUS* was barely detectable by histochemical assay (Fig. 5b). These differences were also conserved in the following developmental phases: at 6 dpg for *pVvWOX13C_CH::GUS* blue staining was localised in few cells in cotyledons and roots with no signal in young leaves (Fig. 5c–e), while for *pVvWOX13C_CS::GUS* the blue signals were much more extended in cotyledons, hypocotyls and roots (Fig. 5m–o). In mature organs at 14 and 21 dpg, GUS activity decreased for both constructs (Fig. 5f–i, p–s); in plants transformed with *pVvWOX13C_CH::GUS,* no activity was detected in flowers and siliques (Fig. 5h,j,k), and for *pVvWOX13C_CS::GUS,* only weak signals were detected in young sepals (Fig. 5r,t,u). These expression levels were confirmed by qRT-PCR, with higher expression induced by *pVvWOX13C_CS::GUS* in young organs, particularly at 6 dpg (Fig. 5a). In addition, also in the presence of PGRs, *pVvWOX13C_CS* induced more *GUS* expression than *pVvWOX13C_CH* in the leaves and roots of *Arabidopsis* (Supplementary Fig. S5b). As reported above for *VvWOX6*, the point mutations identified in these promoters (Supplementary Figs S1 and S2) were likely responsible for the different transcriptional regulation of *VvWOX13C* in CH and CS^20^ (Fig. 1).

*GUS* expression levels induced in *Arabidopsis* by the six promoters isolated from *VvWOX4, VvWOX6* and *VvWOX13C* were significantly lower than those induced by the constitutive p35S (Supplementary Fig. S6). Analysing the relative expression levels, the activity of the *VvWOX* promoters appeared concentrated in young plantlets, whereas in mature tissues (e.g. roots at 21 dpg) the expression was generally low, as demonstrated by the expression induced by *pVvWOX6_CH* resulting at least 1000 times lower than for *p35S::GUS* (Supplementary Fig. S6). These indications could be crucial for the application of these promoters in functional studies in grapevine, in substitution of the traditional p35S^4^.

### Characterisation of promoters isolated from *VvWOX* genes in grapevine embryos

In order to extend the characterisation of *VvWOX* promoters in grapevine, a transient transformation of somatic embryos was used. This approach has proven effective in grapevine^4^,^40^ and it represents a good alternative to stable genetic transformation that is still a time consuming process in grapevine^21^.

**Figure 6:**
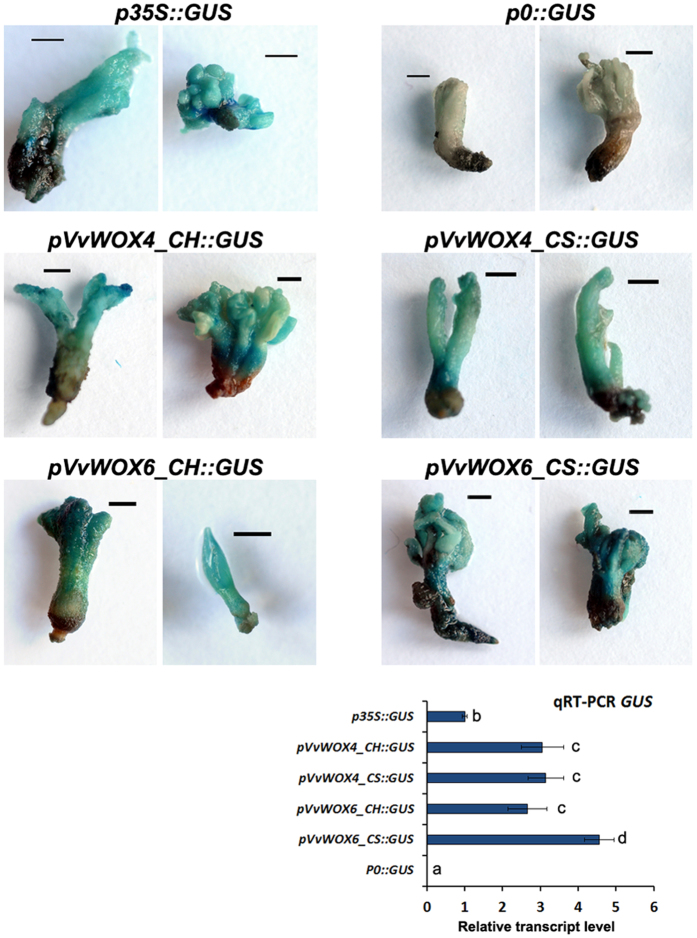
Histochemical and qRT-PCR analysis of GUS activity in transiently transformed grapevine somatic embryos. Data shown are representative of three independent experiments using the constructs *pVvWOX4_CH::GUS, pVvWOX4_CS::GUS, pVvWOX6_CH::GUS* and *pVvWOX6_CS::GUS. p35S::GUS* and *p0::GUS* were used respectively as positive and negative controls of agroinfiltration. Lowercase letters denote significant differences attested by Tukey’s HSD test (P < 0.05). Data are expressed as means ± SE (n = 3). Size bar = 1 mm.

**Figure 7:**
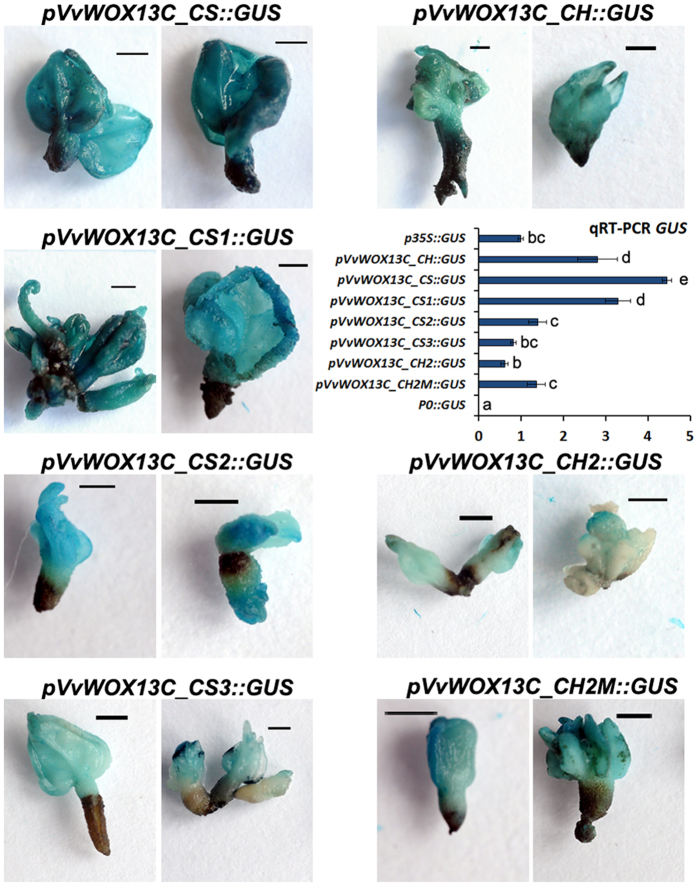
Histochemical and qRT-PCR analysis of GUS activity in transiently transformed grapevine somatic embryos. Data shown are representative of three independent experiments using the complete and truncated constructs derived by *pVvWOX13C_CH* and *pVvWOX13C_CS*. p35S::GUS and p0::GUS were used respectively as positive and negative controls of agroinfiltration. Lowercase letters denote significant differences attested by Tukey’s HSD test (P < 0.05). Data are expressed as means ± SE (n = 3). Size bar = 1 mm.

**Figure 8:**
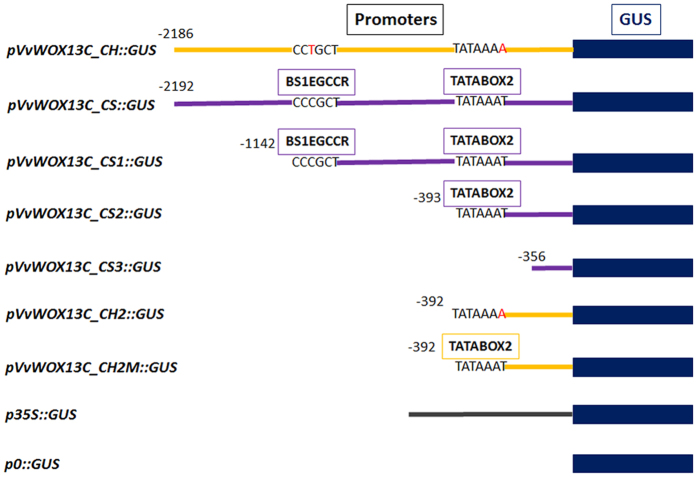
Schematic representation of truncated versions of promoters derived by *pVvWOX13C_CH* and *pVvWOX13C_CS* and fused with *GUS* gene in the expression vector pMDC164.

For the promoters isolated from *VvWOX4* and *VvWOX6,* the histochemical and qRT-PCR experiments carried out on transiently transformed embryos substantially confirmed the results reported above for *Arabidopsis*. No differences in expression levels were observed for the *pVvWOX4_CH::GUS* and *pVvWOX4_CS::GUS* constructs, while *pVvWOX6_CS::GUS* induced high GUS activity in comparison to *pVvWOX6_CH::GUS* (Fig. 6). In addition, for the promoters of *VvWOX13C* the transient transformation confirmed a higher capacity of the sequence isolated from CS to activate the *GUS* transcription in somatic embryos (Fig. 7). In order to understand the role in the gene regulation of some point mutations associated with putatively TFBSs present only in *pVvWOX13C_CS*, truncated versions of *pVvWOX13C_CH* and *pVvWOX13C_CS* were analysed (Fig. 8). Three truncated versions of *pVvWOX13C_CS* were used to produce promoter::*GUS* fusion constructs: i) *pVvWOX13C_CS1::GUS*, containing a fragment of 1142 bp with the TFBS ‘BS1EGCCR’ (Supplementary Fig. S2) involved in vascular expression of the cinnamoyl CoA reductase gene^41^ and present in a single copy only in the sequence isolated from CS; ii) *pVvWOX13C_CS2::GUS* containing a fragment of 393 bp with a TATA-box (Supplementary Fig. S2) absent in the sequence of CH; iii) *pVvWOX13C_CS3::GUS* containing a fragment of 356 bp proximal to the 5′-end of the gene and with seven point mutations between CH and CS (Fig. 8, Supplementary Fig. S1). For the promoter *pVvWOX13C_CH*, two truncated versions were generated and used in promoter::*GUS* fusion constructs: i) *pVvWOX13C_CH2::GUS*, containing a fragment of 392 bp, homologous to *pVvWOX13C_CS2*, but devoid of TATA-box and with nine point mutations (Fig. 8, Supplementary Fig. S1); ii) *pVvWOX13C_CH2M::GUS*, containing the same fragment of *pVvWOX13C_CH2* but with a mutation in a single base (the A was mutated to T at position -387) to generate a functional TATA-box (Fig. 8). The three shortened versions of *pVvWOX13C_CS* induced a progressive reduction of *GUS* expression (Fig. 7), suggesting that the deleted regions contained important TFBSs involved in gene regulation, even in the more distal regions beyond the position -1142 (Fig. 8, Supplementary Fig. S1). The construct *pVvWOX13C_CS2::GUS* induced a twofold *GUS* expression compared with its homologous *pVvWOX13C_CH2::GUS* cloned from CH. However, a single mutation generating a functional TATA-box (*pVvWOX13C_CH2M::GUS*) was enough to bring the *GUS* expression to the same levels detected for *pVvWOX13C_CS2::GUS* (Figs 7and 8). Then, of the nine point mutations present in *pVvWOX13C_CH2* (Supplementary Fig. S1), only the one at position -387 associated to a TATA-box was necessary to significantly increase the expression induced by the proper sequence isolated from CH (Fig. 7).

## Conclusion

In recent years, a substantial amount of information has been collected on grapevine genome structure and genetic variation within cultivars^42^,^43^,^44^. However, little is known about how these sequence variations influence gene expression and phenotype in different cultivars. The family of *WOX* genes is a class of transcription factors that, in *V. vinifera,* showed different transcription levels in two different cultivars, CH and CS^20^ (Fig. 1). We hypothesised that transcriptional regulation would be different for these genes. In the present work, the analyses of the regions proximal to the transcription start site of *VvWOX1* and *VvWOX9* did not show substantial differences in nucleotides and TFBSs between CH and CS, suggesting that other regulatory regions and/or epigenetic changes^45^ are likely responsible for the transcriptional differences previously reported^20^. Similarly, the same conclusion can be drawn for *pVvWOX4_CH* and *pVvWOX4_CS* after the analyses in *Arabidopsis* and grapevine somatic embryos. Although *pVvWOX4_CS* is characterised by a long deletion of 60 bp at about 700 bp proximal to the transcription start site of the gene, apparently this did not influence the transcription and no difference was detected in the transcriptional levels induced by *pVvWOX4_CH*. Recently, an increasing number of computational approaches have been developed to evaluate DNA sequences regulating the transcription of many genes^46^,^47^. However, our results confirm that a purely computational evaluation of regulatory elements involved in the gene modulation in some cases can be misleading, and a functional assessment is often preferable or necessary, especially for the evaluation of a limited number of sequences.

*pVvWOX13C_CS* and *pVvWOX6_CS* induced a significantly higher expression of *GUS* compared to sequences cloned from CH in both the system adopted, the stable transformation of *Arabidopsis* and the transient expression in grapevine. Interestingly, these differences were linked to some mutations in proximal regulatory regions. In particular, we demonstrated that a TATA-box, present only in *pVvWOX13C_CS* at -393 bp to the transcription start site of the gene, was pivotal for the transcriptional regulation of *VvWOX13C*.

The understanding of *VvWOX* regulation in different cultivars of grapevine, which are characterised by different potentials to form embryogenic tissues, would be useful for understanding and improving this regenerative process, fundamental to prospective large-scale applications in new “Sustainable Biotechnology”, i.e. cis-genesis and genome editing in grapevine^6^,^48^. Indeed, the characterisation of *VvWOX* promoters has provided new information about tissue- and time-specific promoters useful for functional studies and as an alternative to traditional constitutive promoters in cis-genetic approaches. In addition, these results also provide interesting information for genome editing in grapevine^7^,^49^, suggesting that point mutations in regulatory sequences could be determinant for increasing or decreasing gene transcription.

## Methods

### Plant materials

The plant material for the expression analysis of *VvWOX* genes in grapevine was collected in 2015 in an experimental vineyard located in the Piedmont (Northwestern Italy). Vines were trained to a vertical trellis with Guyot pruning; conventional agronomic management was regularly applied in the vineyard. Samples were collected from *V. vinifera* ‘Chardonnay’ (CH) and ‘Cabernet Sauvignon’ (CS), both grafted onto rootstock 1103 Paulsen (*V. rupestris* x V*. berlandieri*), from different organs and in different phenological phases according to the E-L System modified by Coombe^50^: shoot apexes, young leaves, tendrils and flowers (E-L17, May 2015), mature leaves (E-L27, June 2015), green berry at pea size (E-L31, July 2015), berries at harvest and seeds (E-L38, September 2015). The roots were collected from three-year-old greenhouse-grown potted plants. For each cultivar, samples from three plants were pooled to form a biological replicate and immediately frozen in liquid nitrogen (in total three independent biological replicates). Pools were stored at −80 °C until molecular analyses.

Somatic embryogenesis from immature anthers of CH and development of somatic embryos were induced following the protocols reported previously in Gambino *et al*.^20^.

### Promoter isolation, sequencing and construction of transformation vectors

Promoter sequences (~2000 bp) proximal to transcription start sites of the genes *VvWOX1, VvWOX4, VvWOX6, VvWOX9* and *VvWOX13C*^20^ were identified into the grapevine genome PN40024^33^. The DNA extracted from leaves of CH and CS using a Plant/Fungi DNA Isolation kit (Norgen Biotek Corp.) was amplified by PCR using specific primers for each promoter (Supplementary Table S3). The PCR reaction mix (50 μl) contained 100 ng of DNA, 0.3 mM dNTPs, 0.3 μM each primer, 1 mM MgSO_4_ and 1 unit of High Fidelity Taq polymerase (Platinum™ Pfx DNA Polymerase, Invitrogen). Cycling conditions for PCRs consisted of initial denaturation at 94 °C for 5 min, followed by 35 cycles of 94 °C for 15 s, 50 °C for 30 s, and 68 °C for 3 min. Products were analysed by electrophoresis on 1% agarose gels buffered in TBE (45 mM Tris-borate, 1 mM EDTA) and visualised by UV-light after staining with ethidium bromide. Amplified products were gel-purified by the Wizard^^®^^ SV Gel and PCR Clean-Up System (Promega) and cloned into the pDONR™/Zeo plasmid (Invitrogen) following the Gateway^^®^^ Technology^39^ to produce Entry Clones using the Gateway^^®^^ BP Clonase^^®^^ II enzyme (Invitrogen). Plasmid DNA was isolated by the Wizard Plus SV Minipreps DNA Purification System (Promega) following the Promega protocol, and sequenced using M13 primers (forward and reverse) and internal primers specific to each promoter (Supplementary Table S3) in order to sequence the entire regions. Products were sequenced using Big-Dye Terminator v1.1 Cycle Sequencing kit (Applied Biosystems), following the manufacturer’s instructions. PCR products were purified using an AutoSeq G-50 Dye Terminator Removal kit (GE Healthcare) and analysed using a 3130 Genetic Analyzer capillary sequencer (Applied Biosystems).

The nucleotide sequences of *pVvWOX* were aligned with the Clustal MUSCLE software (http://www.ebi.ac.uk/Tools/msa/muscle/) using default settings. The phylogenetic analysis based on the Neighbor-Joining (NJ) method was carried out using MEGA7 software^51^. The significance of each node was tested using 1000 bootstrap replicates. The TFBSs present in the promoter sequences and their corresponding TFs were analysed by The Plant Promoter Analysis Navigator (PlantPAN 2.0; http://PlantPAN2.itps.ncku.edu.tw)^36^.

The six promoters *pVvWOX4_CH, pVvWOX4_CS, pVvWOX6_CH, pVvWOX6_CS, pVvWOX13C_CH* and *pVvWOX13C_CS* inserted in pDONR™/Zeo were transferred to the destination vector pMDC164^39^ (https://www.arabidopsis.org/servlets/TairObject?type=vector&id=501100124) for the production of promoter::*GUS* fusion constructs using the Gateway^^®^^ LR Clonase^^®^^ II enzyme (Invitrogen) (Supplementary Fig. S3). The promoters were inserted between the recombination sites *attR1* and *attR2* localized immediately at 5′ of *gusA* gene in the plasmid pMDC164, which contains the Kanamycin resistance for the bacterial selection and the Hygromycin resistance for the selection in plant. The truncated version of the promoters *pVvWOX13C_CH* and *pVvWOX13C_CS* (Fig. 8) were amplified from DNA extracted from CH and CS using specific primers (Supplementary Table S3), sequenced and inserted in pMDC164 as described above. The constitutive p35S promoter was amplified by PCR from the plasmid pGA643 (GenBank accession: AY804024), introduced in pMDC164 and used as positive control for *GUS* expression. All the constructs were inserted in the *Agrobacterium tumefaciens* strain GV3101 by the freeze-thaw method^52^.

### Arabidopsis transformation

The promoter::*GUS* fusion constructs (Supplementary Fig. S3) were used for the transformation of *Arabidopsis thaliana*, ecotype Columbia (Col-0), using the ‘floral dip’ method^53^. Wild type seeds were distributed on the surface of the moistened potting soil, pots were placed at 4 °C for 48 h and then transferred to a growth room with a 16 h photoperiod at 24 °C. After 4 to 6 weeks, when inflorescences were composed of many unopened floral buds, plants were dipped upside down in the *Agrobacterium* inoculum obtained resuspending the bacterial cells immediately before use in a liquid infiltration medium [0.5X Murashige & Skoog (MS) basal salt mixture^54^, 5% sucrose, pH 5.7 and 0.005% Silwet L-77]. The plants were transferred to the growth room until the harvest of seeds. For the screening of transformants, seeds sterilised with sodium hypochlorite (1.5% available chlorine) were distributed in Petri dishes on a selection medium containing 1X MS, 3.0% sucrose, 0.6% agar, pH 5.7, and 30 mg/L of Hygromycin, added after sterilisation at 121 °C for 10 min. Resistant seedlings that survived to selection were transferred to pots and to the growth room for the production of T2 seeds. In the following generations, the seeds sowed *in vitro* under selective conditions were used to determine the ratio of resistant to sensitive seedlings and to calculate the number of segregating T-DNA loci. For each construct, three independent transgenic lines, containing a single insertion in homozygosis, were analysed. qRT-PCR and GUS histochemical assays were carried out on T4 seedlings cultured *in vitro* and collected at 3, 6, 14 and 21 days post germination (dpg). Flowers and siliques were collected from the same plants after the transfer to pots and growth room.

The effects of PGRs on *VvWOX* promoters were studied on *Arabidopsis* seedlings at 6 dpg, transferring the plantlets onto the same selection medium described above with the addition of 4.5 μM 2,4-D and 8.9 μM BA^20^. After 8 days of culture, control seedlings and seedlings subjected to PGRs were collected and analysed by qRT-PCR.

### Transient expression assays in grapevine embryos

Somatic embryos of CH were subjected to agroinfiltration following the protocol of Xu *et al*.^55^ using the same promoter::*GUS* fusion constructs inserted into *Arabidopsis* (Supplementary Fig. S3) and the truncated version of *pVvWOX13C* (Fig. 8). After an overnight culture of *Agrobacterium*, the bacteria were collected by centrifugation, resuspended in an infiltration medium (10 mM MES pH 5.6, 10 mM MgCl_2_, 2% (w/v) sucrose and 150 μM of acetosyringone) and incubated at 28 °C for 3 h to reach a final concentration of 0.6 OD_600_. The embryos were placed in the bacterial solution in a desiccator and subjected to vacuum (0.07 MPa) for 1 h, during which the vacuum was quickly released and restored several times to facilitate the entry of the bacterial suspension into the embryo tissues. The infiltrated embryos were then washed in sterile water, placed over a wet Whatman paper in a Petri dish and incubated in a growth chamber at 25 °C for three days before qRT-PCR and GUS histochemical assays. For each construct, three independent experiments, used as independent biological replicates, were carried out.

### qRT-PCR analysis

Total RNA was extracted from grapevine tissues, agroinfiltrated somatic embryos and plantlets of *Arabidopsis* using the Spectrum™ Plant Total RNA extraction kit (Sigma Aldrich). Total RNA quantity was checked using a NanoDrop 1000 spectrophotometer (Thermo Fisher Scientific) and treated with DNase I (Invitrogen) in accordance with the manufacturer’s instructions. For each biological replicate, first-strand cDNA was synthesised from a starting quantity of 1 μg of total RNA using the High Capacity cDNA Reverse Transcription kit (Applied Biosystems) according to the manufacturer’s instructions. Target-specific primers (Supplementary Table S3) were designed using Primer Express^^®^^ software (v3.0, Applied Biosystems). Reactions were carried out using Power SYBR^^^®^^^ Green PCR Master Mix (Applied Biosystems) as reported in Gambino *et al*.^20^. Three technical replicates were run for each biological replicate, and the expression of transcripts was quantified after normalisation to two housekeeping genes: ubiquitin (*VvUBI*) and actin (*VvACT*) for grapevine tissues^20^, *AtSAND* and *AtTIP41* for *Arabidopsis* plantlets^56^.

The data were subjected to statistical analysis by one-way analyses of variance (ANOVA) with treatment as the main factor using the SPSS 23.0 statistical software package (SPSS Inc., Cary, NC, USA). Tukey’s HSD test was applied when ANOVA showed significant differences (P < 0.05). The standard error (SE) of all means were calculated.

### GUS histochemical assays

The histochemical staining of *Arabidopsis* transgenic plantlets and grapevine agroinfiltrated embryos was carried out following the protocol of Jefferson^57^. Plant tissues immersed in GUS staining solution [50 mM NaH_2_PO_4_ pH 7.0, 5 mM K_4_Fe(CN)_6·_3H_2_O, 5 mM K_3_Fe(CN)_6_, 0.1% Triton X-100 and 1 mM of 5-bromo-4-chloro-3-indolyl-beta-D-glucuronic acid (X-Gluc, Sigma-Aldrich)] were subjected to vacuum (0.07 MPa) for 5 min and then incubated for 24 h at 37 °C. For *Arabidopsis* plantlets, chlorophyll was removed from the leaves by successive washes with 70% ethanol at room temperature. Imaging was performed using an optical microscope with a 10X objective (Nikon Eclipse 55i, Tokyo, Japan).

## Additional Information

**How to cite this article:** Boccacci, P. *et al*. Cultivar-specific gene modulation in *Vitis vinifera*: analysis of the promoters regulating the expression of WOX transcription factors. *Sci. Rep.* **7**, 45670; doi: 10.1038/srep45670 (2017).

**Publisher's note:** Springer Nature remains neutral with regard to jurisdictional claims in published maps and institutional affiliations.

## Supplementary Material

Supplementary Figures

Supplementary Table S1

Supplementary Table S2

Supplementary Table S3
