# Supplementary material for: Cultivar-specific gene modulation in Vitis vinifera: analysis of the promoters regulating the expression of WOX transcription factors
Source: Sci Rep. 2017 Mar 30;7:45670. doi: 10.1038/srep45670 (PMC5372460; doi:10.1038/srep45670)
Supplement: Supplementary Figures [file srep45670-s1.pdf]

Cultivar-specific gene modulation in *Vitis vinifera*: analysis of the promoters regulating the expression of WOX transcription factors.

Paolo Boccacci, Anita Mela, Catalina Pavez Mina, Walter Chitarra, Irene Perrone, Ivana Gribaudo, Giorgio Gambino

Supplementary Figures

Supplementary Figure S1. Sequence Alignment by Clustal MUSCLE tool (http://www.ebi.ac.uk/Tools/msa/muscle/) of the promoters sequences cloned from 'Chardonnay' (CH) and 'Cabernet Sauvignon' (CS) of the genes *VvWOX1*, *VvWOX4*, *VvWOX6*, *VvWOX9* and *VvWOX13C*. The mutations between the sequences isolated from the two cultivars were underlined in green.

pVvWOX1

```
pVvWOX1_CH      GGGCGTAATGTATGGTTTACAACAAAAGAAAAAGTCTGCAATTTGGATTTTCGGAAAGTG
pVvWOX1_CS      GGGCGTAATGTATGGTTTACAACAAAAGAAAAAGTCTGCAATTTGGATTTTCGGAAAGTG
                ************************************************************

pVvWOX1_CH      AAATACATGTACTCCTAAATCAGATAAGGGGTTCCAAAGCTCACTATATATTTGAACTCA
pVvWOX1_CS      AAATACATGTACTCCTAAATCAGATAAGGGGTTCCAAAGCTCACTATATATTTGAACTCA
                ************************************************************

pVvWOX1_CH      TATTTGCCGGAAAATTTTCAGATTCAATGGAGTCTTTTGTTTGTTGAGAGATAGGGAGTT
pVvWOX1_CS      TATTTGCCGGAAAATTTTCAGATTCAATGGAGTCTTTTGTTTGTTGAGAGATAGGGAGTT
                ************************************************************

pVvWOX1_CH      TTTGTTCTATACTCCTATACTTGTGGAATTTCTGTGAATATCTCATTTTGGCTACCTCTT
pVvWOX1_CS      TTTGTTCTATACTCCTATACTTGTGGAATTTCTGTGAATATCTCATTTTGGCTACCTCTT
                ************************************************************

pVvWOX1_CH      ATTTCTCCATTTTTCCACCGCAAATCGTTGCTATTCTTCTGTAAAGCCTCTCTCACCAAA
pVvWOX1_CS      ATTTCTCCATTTTTCCACCGCAAATCGTTGCTATTCTTCTGTAAAGCCTCTCTCACCAAA
                ************************************************************

pVvWOX1_CH      TCGCCCCCTTGGTGTCTCAACCATCTCTCTTCCCTTCACCATGTGCGTAGAGTAGTACTA
pVvWOX1_CS      TCGCCCCCTTGGTGTCTCAACCATCTCTCTTCCCTTCACCATGTGCGTAGAGTAGTACTA
                ************************************************************

pVvWOX1_CH      GCTTTGGACTTGGGTTTTGAGTCTATCCCCTGGCCCATCTAGGGCATGGAATTTTTGCGT
pVvWOX1_CS      GCTTTGGACTTGGGTTTTGAGTCTATCCCCTGGCCCATCTAGGGCATGGAATTTTTGCGT
                ************************************************************

pVvWOX1_CH      CCTTCTGCCTCTTTGAAGCCCAAGCCTGAGCTGACCCATGACCTAGTTCGCATTCCAACC
pVvWOX1_CS      CCTTCTGCCTCTTTGAAGCCCAAGCCTGAGCTGACCCATGACCTAGTTCGCATTCCAACC
                ************************************************************

pVvWOX1_CH      TTTCTAAATACAGCCAACCTGCATGTCCATATCAATCTTGAAATCCATGGCAGCTTGTGG
pVvWOX1_CS      TTTCTAAATACAGCCAACCTGCATGTCCATATCAATCTTGAAATCCATGGCAGCTTGTGG
                ************************************************************
```

```
pVvWOX1_CH      ATGAGGTGGTAATGTCGTGGAAACTTGTGGATCAGAGCTCTTGAATCTTCTCAAAGCCAT
pVvWOX1_CS      ATGAGGTGGTAATGTCGTGGAAACTTGTGGATCAGAGCTCTTGAATCTTCTCAAAGCCAT
                ************************************************************

pVvWOX1_CH      CTTTCTAGGCTCCAAAATGAGTATGTGGTACAATATTTTTAAAATATATCTATTTACATA
pVvWOX1_CS      CTTTCTAGGCTCCAAAATGAGTATGTGGTACAATATTTTTAAAATATATCTATTTACATA
                ************************************************************

pVvWOX1_CH      TAACAACTATGGCTTTGGAGCGCTTGAGTGGCACTCAAAGCTATCCTCTCATTTTACTAC
pVvWOX1_CS      TAACAACTATGGCTTTGGAGCGCTTGAGTGGCACTCAAAGCTATCCTCTCATTTTACTAC
                ************************************************************

pVvWOX1_CH      TTGCTGGGGTTTTGGTGCAAAAGATTCAATAGATGGTTACACGTACAAGTGAAATGAAAA
pVvWOX1_CS      TTGCTGGGGTTTTGGTGCAAAAGATTCAATAGATGGTTACACGTACAAGTGAAATGAAAA
                ************************************************************

pVvWOX1_CH      AGAGAATGTTGTATGCCTCTACCCTACAAGTGAAGTAGAAATAAATAATTATTGAACAAT
pVvWOX1_CS      AGAGAATGTTGTATGCCTCTACCCTACAAGTGAAGTAGAAATAAATAATTATTGAACAAT
                ************************************************************

pVvWOX1_CH      GATGATATGAAAAAGGGCAGATATAGATGGGCATGTACACTAACGACTCAAGAATTGAAT
pVvWOX1_CS      GATGATATGAAAAAGGGCAGATATAGATGGGCATGTACACTAACGACTCAAGAATTGAAT
                ************************************************************

pVvWOX1_CH      CATTCCTTCATAATGGAGCCATGGGTGGGGGGGTGCTGAAGCTGTTGAAGCTAAGGGGCT
pVvWOX1_CS      CATTCCTTCATAATGGAGCCATGGGTGGGGGGGTGCTGAAGCTGTTGAAGCTAAGGGGCT
                ************************************************************

pVvWOX1_CH      ATGCATACCCTACCTTCATGGACATGGACATCTCAAAAAATGAAATAAGGAGAGAAGAGA
pVvWOX1_CS      ATGCATACCCTACCTTCATGGACATGGACATCTCAAAAAATGAAATAAGGAGAGAAGAGA
                ************************************************************

pVvWOX1_CH      GCAGCAGCAGAGAAGATTCCAAATCAGACCCTGCAAATTTGATAGCACGTAAAGGTGTTA
pVvWOX1_CS      GCAGCAGCAGAGAAGATTCCAAATCAGACCCTGCAAATTTGATAGCACGTAAAGGTGTTA
                ************************************************************

pVvWOX1_CH      TATCATTCTATCACCATAGGTTAACCTAAAGGACAATAATCTATAGGATATAAAACAAAG
pVvWOX1_CS      TATCATTCTATCACCATAGGTTAACCTAAAGGACAATAATCTATAGGGTATAAAACAAAG
                *********************************************** ************

pVvWOX1_CH      CTATATTATTGAAAGGACAAAGGTGTGAATGGAGGGAGAGAGAGAGAGAAGAGGGTAGAAAA
pVvWOX1_CS      CTATATTATTGAAAGGACAAAGGTGTGAATGGAGGGAGAGAGAGAGAGAAGAGGGTAGAAAA
                ************************************************************

pVvWOX1_CH      GGAATGTAATAAAAGAACCGTACAGAGAGAGAGAGGAGGGGAGGAGAAAGAGAGAGAGACAA
pVvWOX1_CS      GGAATGTAATAAAAGAACCGTACAGAGAGAGAGAGGAGGGGAGGAGAAAGAGAGAGAGACAA
                ************************************************************

pVvWOX1_CH      AGGAGAGGGGGTGTGAGAGAGGGGCAGTTCGGATTTAGAATAAGTGTGAAAGGCACGAGAC
pVvWOX1_CS      AGGAGAGGGGGTGTGAGAGAGGGGCAGTTCGGATTTAGAATAAGTGTGAAAGGCACGAGAC
                ************************************************************

pVvWOX1_CH      AGTGAGAGCAACAATAAATGAGAGAAAGAAGGTGATGAGGCTTATGATAACCCTCATGAT
pVvWOX1_CS      AGTGAGAGCAACAATAAATGAGAGAAAGAAGGTGATGAGGCTTATGATAACCCTCATGAT
                ************************************************************

pVvWOX1_CH      CGTCACGTCGTTTTTCATTCGCTTCTCCCGATAACTCCCAGAATTCCACGCTTCCTCTCT
pVvWOX1_CS      CGTCACGTCGTTTTTCATTCGCTTCTCCCGATAACTCCCAGAATTCCACGCTTCCTCTCT
                ************************************************************

pVvWOX1_CH      CTTCTCTTCGCTTCTTCTCCTCCTTGCTTTTTTGCCTTTGCTTTTTGTCCATTTTAGACA
pVvWOX1_CS      CTTCTCTTCGCTTCTTCTCCTCCTTGCTTTTTTGCCTTTGCTTTTTGTCCATTTTAGACA
                ************************************************************

pVvWOX1_CH      CACACACTCTCTCTCTCCCCCCTTTCTTCTCTCTCCTGATATAGCCCAGTCAACCTCATG
pVvWOX1_CS      CACACACTCTCTCTCTCCCCCCTTTCTTCTCTCTCCTGATATAGCCCAGTCAACCTCATG
                ************************************************************
```

```
pVvWOX1_CH      TCTTCCTCACTTTGTTCCATTTTAAACCAACACCATTCTCTTTCTCCCATCCATTGGTCA
pVvWOX1_CS      TCTTCCTCACTTTGTTCCATTTTAAACCAACACCATTCTCTTTCTCCCATCCATTGGTCA
                ************************************************************

pVvWOX1_CH      ATCTTCATCTCTGAAACCCTGAAACTCAAACGCTCTCTATTCGGCTCACCTCTCCCATTT
pVvWOX1_CS      ATCTTCATCTCTGAAACCCTGAAACTCAAACGCTCTCTATTCGGCTCACCTCTCCCATTT
                ************************************************************

pVvWOX1_CH      CAACTAAATCACTTTTCATTACCATCAAATACATACTACTTGTGACCCATTTCTGGTTTC
pVvWOX1_CS      CAACTAAATCACTTTTCATTACCATCAAATACATACTACTTGTGACCCATTTCTGGTTTC
                ************************************************************

pVvWOX1_CH      TCCTTCCAGAGTAAAAGACACACTTGGTGGAGCATTATATATCTACAGTTGTAGACCACC
pVvWOX1_CS      TCCTTCCAGAGTAAAAGACAGACTTGGTGGAGCATTATATATCTACAGTTGTAGACCACC
                ******************** ***************************************

pVvWOX1_CH      TCCACGCCAATCCACCATTGCTTGCTCCATTCCCATTTGATCTGCAACTCAAAAACCTAA
pVvWOX1_CS      TCCACGCCAATCCACCATTGCTTGCTCCATTCCCATTTGATCTGCAACTCAAAAACCTAA
                ************************************************************

pVvWOX1_CH      TTTTCAGCCCAATATCATATATCATATGATTATATGCTAGTTGGGAAACAAAAAACATTC
pVvWOX1_CS      TATTCAGCCCAATATCATATATCATATGATTATATGCTAGTTGGGAAACAAAAAACATTC
                * **********************************************************

pVvWOX1_CH      ATGATTAGACTCTCCTTCTTGTTCATTACTCATCTCTACACCCAAAATCCTTTCCTCTAG
pVvWOX1_CS      ATGATTAGACTCTCCTTCTTGTTCATTACTCATCTCTACACCCAAAATCCTTTCCTCTAG
                ************************************************************
                                                          -1
pVvWOX1_CH      TTTGGTGTTCTTTACCCTCCACCTTCAAAGCAAAGCAACTTACA
pVvWOX1_CS      TTTGGTGTTCTTTACCCTCCACCTTCAAAGCAAAGCAACTTACA
                ********************************************
```

Percentage of identity between the two sequences: 99.8%.

pVvWOX4

```
pVvWOX4_CH      GTGATTGATGGGGGGTGGTGTCATGGAATCTGCTTAAGGTAGGCCAAAACATGGACTCAC
pVvWOX4_CS      GTGATTGATGGGGGGTGGTGTCATGGAATCTGCTTAAGGTAGGCCAAAACATGGACTCAC
                ************************************************************

pVvWOX4_CH      ATCCTATATAATCTCTGGAATGTCAACATTTCTATGATTATCCTAGGCATTCACGTCCAT
pVvWOX4_CS      ATCCTATATAATCTCTGGAATGTCAACATTTCTATGATTATCCTAGGCATTCACGTCCAT
                ************************************************************

pVvWOX4_CH      TTAGAATGACATCTGCCCAATAGAATTGAAGAGGTCATCTTCATTTCAAAGTTACTTTGC
pVvWOX4_CS      TTAGAATGACATCTGCCCAATAGAATTGAAGAGGTCATCTTCATTTCAAAGTTACTTTGC
                ************************************************************

pVvWOX4_CH      TTTTTCTAAGTACACAATTGGGATATAAATTCACCGTGCAGGTTAATTATTACACCAAAA
pVvWOX4_CS      TTTTTCTAAGTACACAATTGGGATATAAATTCACCGTGCAGGTTAATTATTACACCAAAA
                ************************************************************

pVvWOX4_CH      TAACCATACAGGTAATTCTCTAAAAAGGGCTGGCTGTTGTAGTAGTTTCGCTTTCAAATT
pVvWOX4_CS      TAACCATACAGGTAATTCTCTAAAAAGGGCTGGCTGTTGTAGTAGTTTCGCTTTCAAATT
                ************************************************************

pVvWOX4_CH      TTAATTAATCTGTACCATATTATCATCAACAGTTGAGAAGAAATTAACACAGTGTACGCG
pVvWOX4_CS      TTAATTAATCTGTACCATATTATCATCAACAGTTGAGAAGAAATTAACACAGTGTACGCG
                ************************************************************

pVvWOX4_CH      TATCCTTGTGATATCAAGCTGGATGGATTATATGGATATATGTCCCTCATGTCGCTTACT
pVvWOX4_CS      TATCCTTGTGATATCAAGCTGGATGGATTATATGGATATATGTCCCTCATGTCGCTTACT
                ************************************************************

pVvWOX4_CH      CTCTGTAGCTTCTGCATGGAGAGTTCAGAATGAGCTGTTCAGCAAATCAACATCCAAACA
pVvWOX4_CS      CTCTGTAGCTTCTGCATGGAGAGTTCAGAATGAGCTGTTCAGCAAATCAACATCCAAACA
```

```
                    ************************************************************

pVvWOX4_CH          TCACGCTGAGTATTTGTCAGTGTCAATTGCCTTAATTCGTACTATGTTAGGGAAGGAAGG
pVvWOX4_CS          TCACGCTGAGTATTTGTCAGTGTCAATTGCCTTAATTCGTACTATGTTAGGGAAGGAAGG
                    ************************************************************

pVvWOX4_CH          AAGGAAGGAAGGAAGGAAGGAGAAATTATGGGTCACATCAGACTGGCAACCCAATCCTTT
pVvWOX4_CS          AAGGAAGGAAGGAAGGAAGGAGAAATTATGGGTCACATCAGACTGGCAACCCAATCCTTT
                    ************************************************************

pVvWOX4_CH          CGAACAAGTATATTCATATTTACCGGGTGGGAAGGGGGAGAGGAGGATCCATAGCAGGTG
pVvWOX4_CS          CGAACAAGTATATTCATATTTACCGGGTGGGAAGGGGGAGAGGAGGATCCATAGCAGGTG
                    ************************************************************

pVvWOX4_CH          GGTGGATGTTAGTTACAGAACAATCATGCCTGGAGAATACTTGGCTAGATTCACGCATGG
pVvWOX4_CS          GGTGGATGTTAGTTACAGAACAATCATGCCTGGAGAATACTTGGCTAGATTCACGCATGG
                    ************************************************************

pVvWOX4_CH          AGTGCCTTTGATTGCTGATTCAAGGAGGAATAATAATGTTTGAGGTCTGAAATGGCCCCA
pVvWOX4_CS          AGTGCCTTTGATTGCTGATTCAAGGAGGAATAATAATGTTTGAGGTCTGAAATGGCCCCA
                    ************************************************************

pVvWOX4_CH          GTTGAATTTTTTCTTAACTTCTTCTAGATATCAGGTAGGTTCATCAAGAAATTGTGGTTT
pVvWOX4_CS          GTTGAATTTTTTCTTAACTTCTTCTAGATATCAGGTAGGTTCATCAAGAAATTGTGGTTT
                    ************************************************************

pVvWOX4_CH          TTGATTCCTTGGTCATATTGTCCTGTCACTCTCAGAAGAACACCCGACAATCCCTCTTCT
pVvWOX4_CS          TTGATTCCTTGGTCATATTGTCCTGTCACTCTCAGAAGAACACCCGACAATCCCTCTTCT
                    ************************************************************

pVvWOX4_CH          CTTATTCAATCAAAACCCCACATCCCCGTATACCTCCAAAGATCCACGACACAAGATGTG
pVvWOX4_CS          CTTATTCAATCAAAACCCCACATCCCCGTATACCTCCAAAGATCCACGACACAAGATGTG
                    ************************************************************

pVvWOX4_CH          GCTGGAATTGGCCTGTAATGCTAAATGTCAAAGTATACAGATTACATACTGTACAAAAGG
pVvWOX4_CS          GCTGGAATTGGCCTGTAATGCTAAATGTCAAAGTATACAGATTACATACTGTACAAAAGG
                    ************************************************************

pVvWOX4_CH          GTCAGAAGTCATGCTGTCTTGAAACCTTTCCAAACAAAGACGCGAGGGTTTAGGGTCATA
pVvWOX4_CS          GTCAGAAGTCATGCTGTCTTGAAACCTTTCCAAACAAAGACGCGAGGGTTTAGGGTCATA
                    ************************************************************

pVvWOX4_CH          TTTGCGGGGATTGAAGGATTTCAGTCGACCCAAGACTCTCCACATTAGGCCCCCAAGAAA
pVvWOX4_CS          TTTGCGGGGATTGAAGGATTTCAGTCGACCCAAGACTCTCCACATTAGGCCCCCAAGAAA
                    ************************************************************

pVvWOX4_CH          GACGCTCGTATGCGTTAGATGCTATAGAAGGAGAATCGAAAAGGCCACAGAAATTTGCAA
pVvWOX4_CS          GACGCTCGTATGCGTTAGATGCTATAGAAGGAGAATCGAAAAGGCCACAGAAATTTGCAA
                    ************************************************************

pVvWOX4_CH          TAATTTGTGAGAGAGTTTATGAATGCAGCACATGTGTGGTGTATATCTCACTTGATAATT
pVvWOX4_CS          TAATTTGTGAGAGAGTTTATGAATGCAGCACATGTGTGGTGTATATCTCACTTGATAATT
                    ************************************************************

pVvWOX4_CH          GCCAATCAGTGAAGAAGAAGCTGTGATTGCGGTCATCAGGACAACTGTAACAATAGCTTT
pVvWOX4_CS          GCCAATCAGTGAAGAAGAAGC---------------------------------------
                    *********************

pVvWOX4_CH          ACTCAAGACTTGATCATATTGTGTGAAAAGACAATAAGATAAATTTACCCCCATGTAAAA
pVvWOX4_CS          ---------------------TGTGAAAAGACAATAAGATAAATTTACCCCCATGTAAAA
                                         ***************************************

pVvWOX4_CH          TACGTAGAAGATTGAGATATATATATTAGGTGATGAGTGGGAGGGTGAAGAATTTGGAAT
pVvWOX4_CS          TACGTAGAAGATTGAGATATATATATTAGGTGATGAGTGGGAGGGTGAAGAATTTGGAAT
                    ************************************************************

pVvWOX4_CH          ACAAAAATGAGATGAAAAGTGGGGTTGATGGGTTTATACTATTTGGGAAATTGGAGCTAC
pVvWOX4_CS          ACAAAAATGAGATGAAAAGTGAGGTTGATGGGTTTATACTATTTGGGAAATTGGAGCTAC
                    ********************* **************************************
```

```
pVvWOX4_CH      AGTTCCCAAAGAAGGGGATATGGAATTGAGATAGATTTGTTTGTGGGTTTTTTTTTTTTT
pVvWOX4_CS      AGTTCCCAAAGAAGGGGATATGGAATTGAGATAGATTTGTTTGTGGGTTTTTTTTTTTTT
                ************************************************************

pVvWOX4_CH      TTTTTTTCTCCTTTTTCTTTTTTTTCAACATTCTCTACCACTTAAAGACGGCCTTCAGTTC
pVvWOX4_CS      TTTTTCT---CCTTTTCTTTTTTTTCAACATTCTCTACCACTTAAAGACGGCCTTCAGTTC
                ***** *   * ************************************************

pVvWOX4_CH      CAGTCACCATATCCGTATCTTGAAAAGCAAAGAGTTCCCATATTCAAGTTTTGGGATTGG
pVvWOX4_CS      CAGTCACCATATCCGTATCTTGAAAAGCAAAGAGTTCCCATATTCAAGTTTTGGGATTGG
                ************************************************************

pVvWOX4_CH      AGGAATTTTTTTTCATTCTCTTCCCTCCATCGCCCTCTCTGCTCACTTTATAGAGAAAAA
pVvWOX4_CS      AGGAATTTTTTTTCATTCTCTTCCCTCCATCGCCCTCTCTGCTCACTTTATAGAGAAAAA
                ************************************************************

pVvWOX4_CH      GTTAAGAATGAGAGCCAAAGAGGCCACCGAAAAGCATGGACACCTTATCATGAAAGCAAG
pVvWOX4_CS      GTTAAGAATGAGAGCCAAAGAGGCCACCGAAAAGCATGGACACCTTATCATGAAAGCAAG
                ************************************************************

pVvWOX4_CH      AAAAAAACGACCGATTTGACGGGCCAACACACGCATATACACACACCACACGCTCATACA
pVvWOX4_CS      AAAAAAACGACCGATTTGACGGGCCAACACACGCATATACACACACCACACGCTCATACA
                ************************************************************

pVvWOX4_CH      ATATTTCTCATTCTCTCTCTCTCTTTCTCTCTCTCTATCTTTTCTTTTTTGTATGGAAAT
pVvWOX4_CS      ATATTTCTCATTCTCTCTCTCTCTTTCTCTCTCTCTATCTTTTCTTTTTTGTATGGAAAT
                ************************************************************

pVvWOX4_CH      CCTAAGTCAACCTCACCTCATCATTTGGTCTTGGTTCTTCTAAGCACCACCTCATATCCC
pVvWOX4_CS      CCTAAGTCAACCTCACCTCATCATTTGGTCTTGGTTCTTCTAAGCACCACCTCATATCCC
                ************************************************************

pVvWOX4_CH      ACTTTCTTTTTCTTCTATCTCTCTCTCTCATCTTCCATATAATTACCACTCTCTATCATT
pVvWOX4_CS      ACTTTCTTTTTCTTCTATCTCTCTCTCTCATCTTCCATATAATTACCACTCTCTATCATT
                ************************************************************
                          -1
pVvWOX4_CH      CAATCCTCCCCC
pVvWOX4_CS      CAATCCTCCCCC
                ************
```

Percentage of identity between the two sequences: 96.7%.

pVvWOX6

```
pVvWOX6_CH      TGTATACCAGTGGCAACATTCATTAACTGGAGTCTGAAGGAGCAATAGTAACGGGATAAA
pVvWOX6_CS      TGTATACCAGTGGCAACATTCATTAACTGGAGTCTGAAGGAGCAATAGTAACGAGATAAA
                ***************************************************** ******

pVvWOX6_CH      TTTTCCATTGGGTATAAAATTAGGCTTTCTTAATTACCCCTGCAACTATCCTTCTGTGCT
pVvWOX6_CS      TTTTCCATTGGGTATAAAATTAGGCTTTCTTAATTACCCCTGCAACTATCCTTCTGTGCT
                ************************************************************

pVvWOX6_CH      ACCTGATGAAACAAAAGCGAGGGTATCACAAAGCCCAATGACAACAGTCTCCTTTGACCA
pVvWOX6_CS      ACCTGATGAAACAAAAGCGAGGGTATCACAAAGCCCAATGACAACAGTCTCCTTTGACCA
                ************************************************************

pVvWOX6_CH      TGCACGAGGAGACCTTATTGAGCATATACAATTCCATCTACATTTTTTTTTTTTTTTTTT
pVvWOX6_CS      TGCACGAGGAGACCTAATTGAGCATATACAATTCCATCTACA--TTTTTTTTTTTTTTTT
                *************** **************************  ****************

pVvWOX6_CH      TGCTGCTCTTTGAAATGAGCAGTCCATCATTGCAGTACACCAGCTCTTGGAATCCTATAC
pVvWOX6_CS      TGCTGCTCTTTGAAATGAGCAGTCCATCATTGCAGTACACCAGCTCTTGGAATCCTATAC
                ************************************************************
```

```
pVvWOX6_CH      TTGCTTTTTATTTTTAAATTCATTTTACTTCACTGCAATCAGATATCAATCCTTTTATAC
pVvWOX6_CS      TTGCTTTTTATTTTTAAATTCATTTTACTTCACTGCAATCAGATATCAATCCTTTTATAC
                ************************************************************

pVvWOX6_CH      TAGTTTGAATTGCTTGGATTTTTATGTGGAAAAGATCTAAACTGACTGAAATTCTCGAAT
pVvWOX6_CS      TAGTTTGAATTGCTTGGATTTTTATGTGGAAAAGATCTAAACTGACTGAAATTCTCGAAT
                ************************************************************

pVvWOX6_CH      GTTGAAAAATTTAATCTGCCCCAAAACTCAGAAGAGTAAACATTGTTAGCACATGGACAA
pVvWOX6_CS      GTTGAAAAATTTAAACTGCCCTAAAACTCAGAAGAGTAAACATTGTTAGCACATGGACAA
                ************** ****** **************************************

pVvWOX6_CH      AGTTGAGCAGAACTTCTCGACTCAGATGCAGAATCCTTGAAACTGTCTAGTTTGAAAGAA
pVvWOX6_CS      AGTTGAGCAGAACTTCTCAACTCAGATGCAGAATCCTTGAAACTGTCTAGTTTTAAAGAA
                ****************** ********************************** ******

pVvWOX6_CH      CTGCCAGCTTTGAAGTAAGAAGTAAGATGAATTTAACAACATTCCAAAGTCTAGAAATCA
pVvWOX6_CS      CTGCCAGCTTTGAAGTAAGAAGTAAGATGAATTTAACAACATTCCAAAGTCTAGAAATCA
                ************************************************************

pVvWOX6_CH      AAATCATTATGCCTCTAATGCAGAAACAGACCTCCTCTGGAATCACAATTCTCAGCTCCT
pVvWOX6_CS      AAATCATTATGCCTCTAATGCAGAAACAGACCTCCTCTGGAATCACAATTCTCAGCTCCT
                ************************************************************

pVvWOX6_CH      ACATCTGGTGGCAGGTGCTTCATGTATCTTGTCAATCTTGTTGTGTATTCAAACACAGAT
pVvWOX6_CS      ACATCTGGTGGCAGGTGCTTCATGTATCTTGTCAATCTTGTTGTGTATTCAAACACAG--
                **********************************************************  

pVvWOX6_CH      AAATAAAACAATAATCTTCAGGGCTTCCATTTTCCATAGTTCAATTGTTTGAGGCATGAG
pVvWOX6_CS      --ATAAAACAATAATCTTCAGGGCTTCCATTTTCCATAGTTCAATTGTTTCAGGCATGAG
                  ************************************************ *********

pVvWOX6_CH      ACTTGAACCTGAAAACAATGCATCAGATGGGAGAATCTACCTGGCATGCATGGGCCAGTC
pVvWOX6_CS      ACTTGAACCTGAAAACAATGCATCAGATGGGAGAATCTACCTGGCATGCATGGGCCAGTC
                ************************************************************

pVvWOX6_CH      TGGCCACAGTTTCAGGGCATGTACTGGCCACAGCTTGCTTCATAGCAGTAAACATGTACT
pVvWOX6_CS      TGGCCACAGTTTCAGGGCATGTACTGGCCACAGCTTGCTTCATAGCAGTAAACATGTACT
                ************************************************************

pVvWOX6_CH      CAATTTGCCAGTAGTCTGAACATGGCATTGCACTTTTGAATTTATTTCAGACAAAAAATA
pVvWOX6_CS      CAATTTGCCAGTAGTCTGAACATGGCATTGCACTTTTGAATTTATTTCAGACAAAAAATA
                ************************************************************

pVvWOX6_CH      ACAATGTCATGCTCATTTCTTTTCCCCCTTTTTTTTTTTGGGCAAACATATCAACACCTC
pVvWOX6_CS      ACAATGTCATGCTCATTTCTTTTCCCCC-TTTTTTTTTTGGGCAAACATATCAACACCTC
                **************************** *******************************

pVvWOX6_CH      CAGTAAAAATAGAAGCAGAAATTAATAGTGATATTTGTCACTGCCATTATCACTCATGTC
pVvWOX6_CS      CAGTAAAAATAGAAGCAGAAATTAATAGTGATATTTGTCACTGCCATTATCACTCATGTC
                ************************************************************

pVvWOX6_CH      TTTTTTAGGTTTTCTTTTATATTCTCCCCTACCCAAAAAGAAAAGTATGCCTGCTTGTCT
pVvWOX6_CS      TTTTTTAGGTTTTCTTTTATATTCTCCCCTACCCAAAAAGAAAAGTATGCCTGCTTGTCT
                ************************************************************

pVvWOX6_CH      GATAAATAATTTTCCACCCATCTGTACTGAAAGCTCATCACCATATACAGACTGTCCTGG
pVvWOX6_CS      GATAAATAATTTTCCACCCATCTGTACTGAAAGCTCATCACCATATACAGACTGTCCTGG
                ************************************************************

pVvWOX6_CH      ATTCCCCCGGGATACCTTAGGCCATAGACACTTAACTATGAAGAGATGGATCAAAACTCA
pVvWOX6_CS      ATTCCCCCGGGATACCTTAGGCCATAGACACTTAACTATGAAGAGATGGATCAAAACTCA
                ************************************************************

pVvWOX6_CH      TGACCTCTGAAAGTTGAAGTCTTGCAGCTGATATATGTGGATATATTAGTAACCACAACA
pVvWOX6_CS      TGACCTCTGAAAGTTGAAGTCTTGCAGCTGATACATGTGGATATATTAGTAACCACAACA
                ********************************* **************************

pVvWOX6_CH      GATTGTTATAAGTAGAGATTATGATCTATGATGTCTAGATTCGCTATATGTTCCGTGCAT
```

```
pVvWOX6_CS       GATTGTTATAAGTAGAGATTATGATCTATGATGTCTAGATTCGCTATATATTCCGTGCAT
                 ************************************************* **********

pVvWOX6_CH       GGGGTTGCCGACAGCAGTAGCAAGAAAGAGTAGGCATATGGAACCAAAAATATTATCGTC
pVvWOX6_CS       GGGGTTGCCGACAGCAGTAGCAAGAAAGAGTAGGCATATGGAACCAAAAATATTATCGTC
                 ************************************************************

pVvWOX6_CH       CCTCAATAATTTTGAATATGAGTAAAGCAAGGCATGCATAATTCAGCCTCTAAATGTCTA
pVvWOX6_CS       CCTCAATAATTTTGAATATGAGTAAAGCAAGGCATGCATAATTCAGCCTCTAAATGTCTA
                 ************************************************************

pVvWOX6_CH       ATCCACAGGTTGGCCAAAGAGGACATTTTTGTTCCCATGTGTTCCATGTCCTAGAGAGGT
pVvWOX6_CS       ATCCACAGGTTGGCCAAAGAGGACATTTTTGTTCCCATGTGTTCCATGTCCTAGAGAGGT
                 ************************************************************

pVvWOX6_CH       TAGGCCATGTATGACCGGTAGCACAAGCTTCACAACATAATGATAACCAGTTGGACCGCA
pVvWOX6_CS       TAGGCCATATATGACCGGTAGCACAAGCTTCACAACATAATGATAACCAGTTGGACCGCA
                 ******** ***************************************************

pVvWOX6_CH       TCCTGATAAAATTTCCAAATTCTATTATCTGCATTTAATTCAACAATAATATGTTCCAGA
pVvWOX6_CS       TCCTGATAAAATTTCCAAATTCTATTATCTGCATTTAATTCAACAATAATATGTTCCAGA
                 ************************************************************

pVvWOX6_CH       ATGATTAGCGGACACAAAAAGGCTAGAAGTCTCCATTTTCAAATGGAACAAAAAAAAA-
pVvWOX6_CS       ATGATTAGCGGACACAAAAAGGCTAGAAGACACCATTTTCAAATGGAACAAAAAAAAAA
                 ***************************** * ***************************

pVvWOX6_CH       -AAAGGGGGGGAGGAAATGAAGGAAAAAGGATAAAGAATGAGAGAGAAAGATGGGGTTGT
pVvWOX6_CS       GAAAAAGGGGGAGGAAATGAAGGAAAAAGGATAAAGAATGAGAGAGAAAGATGGGGTTGT
                  ***  ******************************************************

pVvWOX6_CH       GTCAAGGGTTGAAAGGAACTATACAAAGTGAAGTAGTAATAGTGTAGTGTGGGAGGAGAA
pVvWOX6_CS       GTCAAGGGTTGAAAGGAACTATACAAAGTGAAGTAGTAATAGTGTAGTGTGGGAGGAGAA
                 ************************************************************

pVvWOX6_CH       GAGGCAAAGAAGCAAACAACTACAGGGTCATAATATGGTCACGTCGTTGTTATTCCCCGC
pVvWOX6_CS       GAGGCAAAGAAGCAAACAACTACAGGGTCATAATATGGTCACGTCGTTGTTATTCCCCGC
                 ************************************************************

pVvWOX6_CH       CTTCCAATAACTCCCAGAATTCCACGCTTTGTATGCTCCCCCTCTGCTTTTGCTTTGTCC
pVvWOX6_CS       CTTCCAATAACTCCCAGAATTCCACGCTTTGTATGCTCCCCCTCTGCTTTTGCTTTGTCC
                 ************************************************************

pVvWOX6_CH       TTGTTACTACATACCCCGTCTCTCTCACAATTCATAAATAAGAGACCATGGTCAACCTAC
pVvWOX6_CS       TTGTTACTACATACCCCGTCTCTCTCACAATTCATAAATAAGAGAGCATGGTCAACCTAC
                 ********************************************* **************

pVvWOX6_CH       TATATTCCTTGTTCCCTTTTCACTTTTCTTCTGTTTCAAAACCAGAACTCTTCTTTCTCC
pVvWOX6_CS       TATATTCCTTGTTCCCTTTTCACTTTTCTTCTGTTTCAAAACCAGAACTCTTCTTTCTCC
                 ************************************************************

pVvWOX6_CH       ATTTAATCTCTCATTCTTTCTTGTCCACTTTACACCTCTGAAGCTCTCAACG-CCCCTCT
pVvWOX6_CS       ATTTAATCTCTCATTCTTTCTTGTCCACTTTACACCTCTGAAGCTCTCAACGCCCCCTCT
                 **************************************************** *******
                                                                        -1
pVvWOX6_CH       TCTTGTCCACTCTACTACCCATTTCCCTACGAAGCACACTCTTAGTACCATGTGGACG
pVvWOX6_CS       TCTTGTCCACTCTACTACCCATTTCCCTACGAAGCACACTCTTAGTACCATGTGGACG
                 **********************************************************
```

Percentage of identity between the two sequences: 98.8%.

pVvWOX9

```
pVvWOX9_CH       CCTTTCTCTCTGACTTTATTGCCTCCATCTTCACATGCACTCCCCCCTTTTCTTTTTTCT
pVvWOX9_CS       CCTTTCTCTCTGACTTTATTGCCTCCATCTTCACATGCACTCCCCCCTTTTCTTTTTTCT
```

```
                    ************************************************************

pVvWOX9_CH          CTTCTTTATTCACTCGATATATGTCACATTTCACCGGTTAATTCCTTATTGCTATGTCGC
pVvWOX9_CS          CTTCTTTATTCACTCGATATATGTCACATTTCACCGGTTAATTCCTTATTGCTATGTCGC
                    ************************************************************

pVvWOX9_CH          CGACATACGTCCATGATCTAATTCCATTATGCTACAAATCAAGAAGGGATTAATTTCATA
pVvWOX9_CS          CGACATACGTCCATGATCTAATTCCATTATGCTACAAATCAAGAAGGGATTAATTTCATA
                    ************************************************************

pVvWOX9_CH          ATGGCTTGAACTAAAGAACTAGAAAGTGCACGACTAGTTAAAAAAAGCCTTCTCCCCCGC
pVvWOX9_CS          ATGGCTTGAACTAAAGAACTAGAAAGTGCACGACTAGTTAAAAAAAGCCTTCTCCCCCGC
                    ************************************************************

pVvWOX9_CH          TACATGCAACACAAATAACAGGCACGTAGCAAGAGGCAACATGCTCGGCCACCACCACCA
pVvWOX9_CS          TACATGCAACACAAATAACAGGCACGTAGCAAGAGGCAACATGCTCGGCCACCACCACCA
                    ************************************************************

pVvWOX9_CH          ACAATTCCATTGACTATAACATCAACTTTTTCTTAAAGGTTGAATTTTTAGTCTTTCACA
pVvWOX9_CS          ACAATTCCATTGACTATAACATCAACTTTTTCTTAAAGGTTGAATTTTTAGTCTTTCACA
                    ************************************************************

pVvWOX9_CH          TAAATGATTAACATTTGTTCTCACAAAAAAATGTGATCAGCCTACATGGTACCAGTACCAT
pVvWOX9_CS          TAAATGATTAACATTTGTTCTCACAAAAAAATGTGATCAGCCTACATGGTACCAGTACCAT
                    ************************************************************

pVvWOX9_CH          GTCCACACCCTACCTGAAATTTCACGCCACACCAATATTTGCCTCCGCATACCTATGTCA
pVvWOX9_CS          GTCCACACCCTACCTGAAATTTCACGCCACACCAATATTTGCCTCTGCATACCTATGTCA
                    ********************************************* **************

pVvWOX9_CH          CTTTTATAACATTCACTACTGCACTTGTTCATCTGCAATATTAATGTAGCTTAGTTTGAA
pVvWOX9_CS          CTTTTATAACATTCACTACTGCACTTGTTCATCTGCAATATTAATGTAGCTTAGTTTGAA
                    ************************************************************

pVvWOX9_CH          ATTTTTAAATGATTGAAGAAATTGGCCTCCACCGCCATCAAATTGATGTCGTTGCCCATA
pVvWOX9_CS          ATTTTTAAATGATTGAAGAAATTGGCCTCCACCGCCATCAAATTGATGTCGTTGCCCATA
                    ************************************************************

pVvWOX9_CH          ACCACAATTGTGCCATCACCATCATCAGCTCCATCTCTATCTCTAGCGGCATTTTGAGTG
pVvWOX9_CS          ACCACAATTGTGCCATCACCATCATCAGCTCCATCTCTATCTCTAGCGGCATTTTGAGTG
                    ************************************************************

pVvWOX9_CH          TCCCATTGACTTGTAATCATCTGCTTTACCAAAATTTTCTCCTCACAAAATGTTTAAATT
pVvWOX9_CS          TCCCATTGACTTGTAATCATCTGCTTTACCAAAATTTTCTCCTCACAAAATGTTTAAATT
                    ************************************************************

pVvWOX9_CH          TGTGGGTTCAAACTCATCTCGATGTGAAAACTTTGAAAACGAGGAAGAGGTCTTAATGAT
pVvWOX9_CS          TGTGGGTTCAAACTCATCTCGATGTGAAAACTTTGAAAACGAGGAAGAGGTCTTAATGAT
                    ************************************************************

pVvWOX9_CH          TTTTCCTGATTAGGAGACGACTGAAAGACCAGCTAATAGGAGTCAACCAAACAATTAATT
pVvWOX9_CS          TTTTCCTGATTAGGAGACGACTGAAAGACCAGCTAATAGGAGTCAACCAAACAATTAATT
                    ************************************************************

pVvWOX9_CH          GATTGAAAAAAATATTCAAATATAACAACATTACTGAATTTTAATATATAGTACTTCATTA
pVvWOX9_CS          GATTGAAAAAAATATTCAAATATAACAACATTACTGAATTTTAATATATAGTACTTCATTA
                    ************************************************************

pVvWOX9_CH          ATCCTACTCGTTTCATAAGTCCTTCACCAATATTCGTTTGATAGGGTTAATATCCCACTG
pVvWOX9_CS          ATCCTACTCGTTTCATAAGTCCTTCACCAATATTCGTTTGATAGGGTTAATATCCCACTG
                    ************************************************************

pVvWOX9_CH          TTTTATTGGTAGTACCATTTGATAAAAAAAAATTGCTATTTCTTGTCGCAACATTTACAAT
pVvWOX9_CS          TTTTATTGGTAGTACCATTTGATAAAAAAAAATTGCTATTTCTTGTCGCAACATTTACAAT
                    ************************************************************

pVvWOX9_CH          AAAGCTAGTAGAACAAATTGAGAAAGGGGAGTGACATTTTTCTCATTTAATTGGATTTA
pVvWOX9_CS          AAAGCTAGTAGAACAAATTGAGAAAGGGGAGTCACATTTTTCTCATTTAATTGGATTTA
                    ******************************** ***************************
```

```
pVvWOX9_CH      ATTAATTTTGATTTTGAA--------------TTTTTTTTTTGTTTAAGGAAAGAAAATA
pVvWOX9_CS      ATTAATTTTGATTTTGAATTTTTTTTTTTTTTTTTTTTTTTTGTTTAAGGAAAGAAAATA
                ******************              ****************************

pVvWOX9_CH      AAAGAAGAGGAAGAAGAAGAGGCCCTCGAGTCAATCCAAAGCCCACCTTTGCCCAAAATA
pVvWOX9_CS      AAAGAAGAGGAAGAAGAAGAGGCCCTCGAGTCAATCCAAAGCCCACCTTTGCCCAAAATA
                ************************************************************

pVvWOX9_CH      AACCAAACTCGCAATTACTGACCCACCACCTCTTTCCAATCAAAACCCTACAAGGCTCCT
pVvWOX9_CS      AACCAAACTCGCAATTACTGACCCACCACCTCTTTCCAATCAAAACCCTACAAGGCTCCT
                ************************************************************

pVvWOX9_CH      TGACTTTCAAACACCCCCACCCCCATTTATTCCTGCAACAACCTCTTCCATTAGAATAAT
pVvWOX9_CS      TGACTTTCAAACACCCCCACCCCCATTTATTCCTGCAACAACCTCTTCCATTAGAATAAT
                ************************************************************

pVvWOX9_CH      CCCACACTTATATCTACCCCTTCACCCTCCCATTAATACCACTGCAAGCAAACCAAAGTC
pVvWOX9_CS      CCCACACTTATATCTACCCCTTCACCCTCCCATTAATACCACTGCAAGCAAACCAAAGTC
                ************************************************************

pVvWOX9_CH      ATGAGCAAGGGAATCCTCCCCATGGAAAACAATGAATAGGAGACAAACTCATCAGAGCAG
pVvWOX9_CS      ATGAGCAAGGGAATCCTCCCCATGGAAAACAATGAATAGGAGACAAACTCATCAGAGCAG
                ************************************************************

pVvWOX9_CH      CCCACCTCTCTAAAGCTATAAAACCCACTCCACTCCGCACCACCCCCTTTTGCATTAAGA
pVvWOX9_CS      CCCACCTCTCTAAAGCTATAAAACCCACTCCACTCCGCACCACCCCCTTTTGCATTAAGA
                ************************************************************

pVvWOX9_CH      CCCCTTCTCTGTCTCCTCTGATCAAGATTCTAATCTAGCCCCAGCATATATTGCTTTCCA
pVvWOX9_CS      CCCCTTCTCTGTCTCCTCTGATCAAGATTCTAATCTAGCCCCAGCATATATTGCTTTCCA
                ************************************************************

pVvWOX9_CH      TGTTCCCTTTGCTTCTCGTCCGGGGGAATTGGCTTGTACTTTGAGTAGTCCACAAGCATT
pVvWOX9_CS      TGTTCCCTTTGCTTCTCGTCCGGGGGAATTGGCTTGTACTTTGAGTAGTCCACAAGCATT
                ************************************************************

pVvWOX9_CH      TCAGTTTTAATGCTAAACTGTAAGGAAGGGATGGGAGTGTACTCTCCTTCTTACCTTTAT
pVvWOX9_CS      TCAGTTTTAATGCTAAACTGTAAGGAAGGGATGGGAGTGTACTCTCCTTCTTACCTTTAT
                ************************************************************

pVvWOX9_CH      ATTTACTTTCATTTCCTTTCTTGTTTATCATCTCACAGGCGATTCCTGTTGTGGGTTTTG
pVvWOX9_CS      ATTTACTTTCATTTCCTTTCTTGTTTATCATCTCACAGGCGATTCCTGTTGTGGGTTTTG
                ************************************************************

pVvWOX9_CH      TCAATTTTTTTATTATGGGTTTTATTATTAGTATTATTATTTTTATTTTTATTCTTTGGG
pVvWOX9_CS      TCAATTTTTTTATTATGGGTTTTATTATTAGTATTATTATTTTTATTTTTATTCTTTGGG
                ************************************************************

pVvWOX9_CH      AGATGGGAAAGACTGTTTATTAATTGAAATAAACTGAACACAGGGGTAGGTGAGGAGGCT
pVvWOX9_CS      AGATGGGAAAGACTGTTTATTAATTGAAATAAACTGAACACAGGGGTAGGTGAGGAGGCT
                ************************************************************

pVvWOX9_CH      ATAAGAAGAGATGTGATGTTTGGCTAGAGGCTTTCAGTACCAAAAACAAGAGCAACAAAA
pVvWOX9_CS      ATAAGAAGAGATGTGATGTTTGGCTAGAGGCTTTCAGTACCAAAAACAAGAGCAACAAAA
                ************************************************************

pVvWOX9_CH      GGGCTTTGCTCCGTTGTTGTTGTTTCTCTTCTGGTTGGCTACTTTAGATATTTCATATCT
pVvWOX9_CS      GGGCTTTGCTCCGTTGTTGTTGTTTCTCTTCTGGTTGGCTACTTTAGATATTTCATATCT
                ************************************************************
                                                  -1
pVvWOX9_CH      ACATAGCTGCTGGTCTGCCCTCCCATCATCATCATC
pVvWOX9_CS      ACATAGCTGCTGGTCTGCCCTCCCATCATCATCATC
                ************************************
```

Percentage of identity between the two sequences: 99.2%.

pVvWOX13C

```
pVvWOX13C_CH      CATGGATCTTCATGTGTGGTTTCAGTCATGATGCAGGCCTAACCTTTAGTGAAGCACTAA
pVvWOX13C_CS      CATGGATCTTCATGTGTGGTTTCAGTCATGATGCAGGCCTAACCTTTAGTGAAGCACTAA
                  ************************************************************

pVvWOX13C_CH      TAGAAGGTGGATGTCACTTCACCGCAATTTGACTGAAGCAGGCTAAAAGAGTGAATTAGC
pVvWOX13C_CS      TAGAAGGTGGATGTCACTTCACCGCAATTTGACTGAAGCAGGCTAAAAGAGTGAATTAGC
                  ************************************************************

pVvWOX13C_CH      AGCTCTAGATCCAAAGAAACAGTGGAAAGTAGGTGGAAAAGCTAGAGACAAAAAAGATAT
pVvWOX13C_CS      AGCTCTAGATCCAAAGAAACAGTGGAAAGTAGGTGGAAAAGCTAGAGACAAAAAAGATAT
                  ************************************************************

pVvWOX13C_CH      TGAAAAGAAACATCTTTTGAAGATCTGCCACTAGCTTTAGAAATCTTGTGGAATCCGCAA
pVvWOX13C_CS      TGAAAAGAAACATCTTTTGAAGATCTGCCACTAGCTTTAGAAATCTTGTGGAATCCGCAA
                  ************************************************************

pVvWOX13C_CH      GAAATCGTAGAAAAAATTGTCCCACTTCGGACTATTGGGATAGCTATTTGAGGCTCAACAT
pVvWOX13C_CS      GAAATCGTAGAAAAAATTGTCCCACTTCGGACTATTGGGATAGCTATTTGAGGCTCAACAT
                  ************************************************************

pVvWOX13C_CH      GCCCGTCTCTAATTGTGTAGATTGGGCGTTAGGAGTCATGTTAGGTACACCAAATTGTCC
pVvWOX13C_CS      GCCCGTCTCTAATTGTGTAGATTGGGCGTTAGGAGTCATGTTAGGTACACCAAATTGTCC
                  ************************************************************

pVvWOX13C_CH      AATCCAAGGAAGAAAAACAAGACATACTGACGCCACATATCCCATTTTGAATGGTGTATC
pVvWOX13C_CS      AATCCAAGGAAGAAAAACAAGACATACTGACGCCACATATCCCATTTTGAATGGTGTATC
                  ************************************************************

pVvWOX13C_CH      ACCACCATCCCCACCCCCACTGTAGTCCCCCTCACAATCGAACACTTCCTTCTCCAAACC
pVvWOX13C_CS      ACCACCATCCCCACCCCCACTGTAGTCCCCCTCACAATC-AACACTTCCTTCTCCAAACC
                  *************************************** ********************

pVvWOX13C_CH      CTTAGCAGGAATCCTACTGTGGCCAAACTGTGTAATAAGAAGATTCAGAAAAACACGAGT
pVvWOX13C_CS      CTTAGCAGGAATCCTACTGTGGCCAAACTGTGTAATAAGAAGATTCAGAAAAACACGAGT
                  ************************************************************

pVvWOX13C_CH      TGGGCTCAGAGACAGACCGAGTTATTGAGGGTGGATGGTGAAGATGGAGGAGTACATCTG
pVvWOX13C_CS      TGGGCTCAGAGACAGACCGAGTTATTGAGGGTGGATGGTGAGGATGGAGGAGTACATCTG
                  ***************************************** ******************

pVvWOX13C_CH      CA------CATGCCCATGCCCATGTGCGTCATGTTCCACATCTTCACTTTCAAACAATAT
pVvWOX13C_CS      CACATGCCCATGCCCATGCCCATGTGCGTCATGTTCCACATCTTCACTTTCAAACAATAT
                  **      ****************************************************

pVvWOX13C_CH      AATGATTAATCAGAATAAGGTGAATAGCTATAGTTGAAACTTGAAACCAGAACACTTGTG
pVvWOX13C_CS      AATGATTAATCAGAATAAGGTGAATAGCTATAGTTGAAACTTGAAACCAGAACACTTGTG
                  ************************************************************

pVvWOX13C_CH      AGCCCTGCAGACGCGTCAGAGACCAACTCCCCTCCCCCCATTCTAATTTCAAAAGGGGTT
pVvWOX13C_CS      AGCCCTGCAGACGCGTCAGAGACCAACTCCCCTCCCCCCATTCTAATTTCAAAAGGGGTT
                  ************************************************************

pVvWOX13C_CH      TAATCACATTTAGTCTAATCAATGACCCACCCCATGACCCACCCCAATAGTATAAACACA
pVvWOX13C_CS      TAATCACATTTAGTCTAATCAATGACCCACCCCATGACCCACCCCAATAGTATAAACACA
                  ************************************************************

pVvWOX13C_CH      TCATTAGGTCAAGGACCCTGTTCATTAATCACTCTCCTCTTCGTCTCATCTGCCAAATGC
pVvWOX13C_CS      TCATTAGGTCAAGGACCCTGTTCATTAATCACTCTCCTCTTCGTCTCATCTGCCAAATGC
                  ************************************************************

pVvWOX13C_CH      ATGTAAGTCAAGGGCATCTCTTCTTGTTTCTAAATGACAACTTTCTTATTAAACTCTTTC
pVvWOX13C_CS      ATGTAAGTCAAGGGCATCTCTTCTTGTTTCTAAATGACAACTTTCTTATTAAACTCTTTC
                  ************************************************************

pVvWOX13C_CH      TTCTTTAAAGATTTCTCACTTTGTACAAGGTGGAAAGTTGCATCTTCCCATGTGAGTTTA
```

```
pVvWOX13C_CS        TTCTTTAAAGATTTCTCACTTTGTACAAGGTGGAAAGTTGCATCTTCCCATGTGAGTTTA
                    ************************************************************

pVvWOX13C_CH        TGATTCCATGCCAAGAATTTTGTACTCTTGACCTGCTTCAAATCAATCTACAATACACTG
pVvWOX13C_CS        TGATTCCATGCCAAGAATTTTGTTCTCTTGACCCGCTTCAAATCAGTCTACAATACACTG
                    *********************** ********* *********** **************

pVvWOX13C_CH        GATACATACATTGGATACCCACTCAGTAGCTCAGTAGCTCATGTACATGAAATGTCAACA
pVvWOX13C_CS        GATACATACATTGGATACCCACTCAGTAGCTCAGTAGCTCATGTACATGAAATGTCAACA
                    ************************************************************

pVvWOX13C_CH        TGGGAACCTTAATTCGGCCAAAATTCAAACTCTTGGTGATTGTATATTTTATTAATTAAG
pVvWOX13C_CS        TGGGAACCTTAATTCGGCCAAAATTCAAACTCTTGGTGATTGTATATTTTATTAATTAAG
                    ************************************************************

pVvWOX13C_CH        TAATATAACAAATCTTGTACATTAAAAGTGTAGGTTTGAAATTTAGCAAGGGTGAGAGGC
pVvWOX13C_CS        TAATATAACAAATCTTGTATATTAAAAGTGTAGGTTTGAAATTTAGCAAGGGTGAGAGGC
                    ******************* ****************************************

pVvWOX13C_CH        ACGTGCACGTGGGTAGGGTGGAAATGGAAGAATGAAAGCAGGCGCCATTAAAATCCTGGT
pVvWOX13C_CS        ACGTGCACGTGGGTAGGGTGGAAATGGAAGAATGAAAGCAGGCGCCATTAAAATCCTGGT
                    ************************************************************

pVvWOX13C_CH        AATAACGCCAGAATGGTGCTCCCAACAGCACCTGAATCTGAAGTTTTTCCCATTTATGTA
pVvWOX13C_CS        AATAACGCCAGAATGGTGCTCCCAACAGCACCTGAATCTGAAGTTTTTCCCATTTATGTA
                    ************************************************************

pVvWOX13C_CH        GATTGTCAGACACCCACTGCCCCAAACATTCATTCAGCCATGATCCATTCTCACGCAACC
pVvWOX13C_CS        GATTGTCAGACACCCACTGCCCCAAACATTCTTTCAGCCATGATCCATTCTCACGCAACC
                    ******************************* ****************************

pVvWOX13C_CH        CCATTAATCACCTGCTCCGCCCCCTCTCCATGCCCCACTACTTCCACATATCATTCAGTA
pVvWOX13C_CS        CCATTAATCACCTGCTCCGCCCCCTCTCCATGCCCCACTACTTCCACATATCATTCAGTA
                    ************************************************************

pVvWOX13C_CH        TGTATATTTTTTTATTTCCTAAACCCCACATGGACATGATGGGACGCTTTGCTTTGAATG
pVvWOX13C_CS        TGTATATTTTTTTATTTCCTAAACCCCACATGGACATGATGGGACGCTTTGCTTTGAATG
                    ************************************************************

pVvWOX13C_CH        GGGGCCAATGAACTAAAACGAGGGTACATGGAGTGGGGTGGTGGTGAGGAGCAGAGGATG
pVvWOX13C_CS        GGGGCCAATGAACTAAAACGAGGGTACATGGAGTGGGGTGGTGGTGAGGAGCAGAGGATG
                    ************************************************************

pVvWOX13C_CH        AGGTTTTGATTGAGAGACGTGGGAAGGCGTGGTGTCCCATCGTATCCAGGCCAATTTTAC
pVvWOX13C_CS        AGGTTTTGATTGAGAGACGTGGGAAGGCGTGGTGTCCCATCGTATCCAGGCCAATTTTAC
                    ************************************************************

pVvWOX13C_CH        AAAGACACAAGCCCTGTGTATCTTTGAATATTACTTGCATAATATCGTGATATTTTGAGT
pVvWOX13C_CS        AAAGACACAAGCCCTGTGTATCTTTGAATATTACTTGCATAATATCGTGATATTTTGAGT
                    ************************************************************

pVvWOX13C_CH        TCTGCAATCAAGAATGGTCCAGCTGCATGGCAAAAGTGTAATCTAAGTTCTCAAAGCCCT
pVvWOX13C_CS        TCTGCAATCAAGAATGGTCCAGCTGCATGGCAAAAGTGTAATCTAAGTTCTCAAAGCCCT
                    ************************************************************

pVvWOX13C_CH        TATAAAAAAATATTAAAACTTATGTGTTGTTTTTATTTTTATTTTTATTTTTATTTTATTT
pVvWOX13C_CS        TATAAATAAATATTAAAGCTTATGTGTTGTTTTTATTTTTAATTTAATTTAATTTTATTT
                    ****** ********** *********************** *** **** *********

pVvWOX13C_CH        TATTTTATGAGAATTTCAGAAATAAAAAACTAAAAATAAAAA-AATAATAAAAATTAATA
pVvWOX13C_CS        TATTTTTTGAGAATTTGAGAAATAAAAAACTAAAAATAAAAATAAAAATAAAAATTAATA
                    ****** ********* ************************* ** **************

pVvWOX13C_CH        TATGGTAGGTTTAAGTGTGAGTGCGTGTGTGTTTTTTTTGGAAGATTGATGTGGGTTGTT
pVvWOX13C_CS        TATGGTAGGTTTAAGTGTGAGTGCGTGTGTGTTTTTTTTGGAAGATTGATGTGGGTTGTT
                    ************************************************************

pVvWOX13C_CH        TTATCATGAACCGAGAAAATGGACGTGTGAAAACGCGCATGAGACACATGCCCATGCTAA
pVvWOX13C_CS        TTATCATGAACCGAGAAAATGGACGTGTGAAAACGCGCATGAGACACATGCCCATGCTAA
```

```
                       ************************************************************

pVvWOX13C_CH           AGTACGCCCCAGGAGGAGAGTACACTAACATACACCATCTTTTTCGTGGTTTCCTTTTTA
pVvWOX13C_CS           AGTACGCCCCAGGAGGAGAGTACACTAACATACACCATCTTTTTCGTGGTTTCCTTTTTA
                       ************************************************************

pVvWOX13C_CH           CCTTTTGGTGTGTGTAAACACTACTCAGTGCTGACTTTGTTAGAGAGATGGAGAGAGAGT
pVvWOX13C_CS           CCTTTTGGTGTGTGTAAACACTACTCAGTGCTGACTTTGTTAGAGAGATGGAGAGAGAGT
                       ************************************************************
                                                      -1
pVvWOX13C_CH           AACGAGAGCGAGAGAGGAGGAGAAGAAGAGAGT
pVvWOX13C_CS           AACGAGAGCGAGAGAGGAGGAGAAGAAGAGAGT
                       *********************************
```

Percentage of identity between the two sequences: 98.9%.

```
                -792                   CAATBOX1 WBOXNTERF3        CAATBOX1 -733
pVvWOX4_CH       GCCAATCAGTGAAGAAGAAGCTGTGATTGCGGTCATCAGGACAACTGTAACAATAGCTTT
pVvWOX4_CS       GCCAATCAGTGAAGAAGAAGC---------------------------------------
                 *********************

               -732 ACTFTPPCA1   CAATBOX1                                -673
pVvWOX4_CH       ACTCAAGACTTGATCATATTGTGTGAAAAGACAATAAGATAAATTTACCCCCATGTAAAA
pVvWOX4_CS       ---------------------TGTGAAAAGACAATAAGATAAATTTACCCCCATGTAAAA
                                      ***************************************

                -592                                                    -533
pVvWOX4_CH       ACAAAAATGAGATGAAAAGTGGGGTTGATGGGTTTATACTATTTGGGAAATTGGAGCTAC
pVvWOX4_CS       ACAAAAATGAGATGAAAAGTGAGGTTGATGGGTTTATACTATTTGGGAAATTGGAGCTAC
                 ********************* **************************************
                                 GTGANTG10

                -492 GT1GMSCAM4                                         -433
pVvWOX4_CH       TTTTTTCTCCTTTTTCTTTTTTTCAACATTCTCTACCACTTAAAGACGGCCTTCAGTTC
pVvWOX4_CS       TTTTTCT---CCTTTTCTTTTTTTCAACATTCTCTACCACTTAAAGACGGCCTTCAGTTC
                 ***** *   * ************************************************
________________________________________________________________________________

                -898                                                    -839
pVvWOX6_CH       GATTGTTATAAGTAGAGATTATGATCTATGATGTCTAGATTCGCTATATGTTCCGTGCAT
pVvWOX6_CS       GATTGTTATAAGTAGAGATTATGATCTATGATGTCTAGATTCGCTATATATTCCGTGCAT
                 ************************************************* **********
                                                          ROOTMOTIFTAPOX1

                -538                          SURECOREATSULTR11         -479
pVvWOX6_CH       ATGATTAGCGGACACAAAAAGGCTAGAAGTCTCCATTTTCAAATGGAACAAAAAAAAA-
pVvWOX6_CS       ATGATTAGCGGACACAAAAAGGCTAGAAGACACCATTTTCAAATGGAACAAAAAAAAAA
                 ***************************** * ***************************
                                                                  DOFCOREZM

                -478                                                    -419
pVvWOX6_CH       -AAAGGGGGGGAGGAAATGAAGGAAAAAGGATAAAGAATGAGAGAGAAAGATGGGGTTGT
pVvWOX6_CS       GAAAAAGGGGGAGGAAATGAAGGAAAAAGGATAAAGAATGAGAGAGAAAGATGGGGTTGT
                  ***  ******************************************************
                 GT1CONSENSUS

                -235                                SURECOREATSULTR11   -179
pVvWOX6_CH       TTGTTACTACATACCCCGTCTCTCTCACAATTCATAAATAAGAGACCATGGTCAACCTAC
pVvWOX6_CS       TTGTTACTACATACCCCGTCTCTCTCACAATTCATAAATAAGAGAGCATGGTCAACCTAC
                 ********************************************* **************
________________________________________________________________________________

                -1174           CACTFTPPCA1 QELEMENTZMZM13  CAATBOX1   -1114
pVvWOX13C_CH     TGATTCCATGCCAAGAATTTTGTACTCTTGACCTGCTTCAAATCAATCTACAATACACTG
pVvWOX13C_CS     TGATTCCATGCCAAGAATTTTGTTCTCTTGACCCGCTTCAAATCAGTCTACAATACACTG
                 ********************** ********* *********** **************
                                                BS1EGCCR

                -393                                          MARTBOX   -334
pVvWOX13C_CH     TATAAAAAAATATTAAAACTTATGTGTTGTTTTTATTTTTATTTTTATTTTATTTTATTT
pVvWOX13C_CS     TATAAATAAATATTAAAGCTTATGTGTTGTTTTTATTTTTAATTTAATTTAATTTTATTT
                 ****** ********** ********************** *** **** *********
                 TATABOX2     TAAAGSTKST1

                -333      EECCRCAH1                          POLASIG3   -274
pVvWOX13C_CH     TATTTTATGAGAATTTCAGAAATAAAAAACTAAAAATAAAAA-AATAATAAAAATTAATA
pVvWOX13C_CS     TATTTTTTGAGAATTTGAGAAATAAAAAACTAAAAATAAAAATAAAAATAAAAATTAATA
                 ****** ********* ************************* ** **************
                                                     EF4MOTIFGM7S
```

Supplementary Figure S2. Parts of the nucleotide sequences of *pVvWOX4, pVvWOX6* and *pVvWOX13C* isolated from ‘Chardonnay’ (CH) and ‘Cabernet Sauvignon’ (CS). The underlined sequences are transcription factor binding sites (TFBSs) present only in CH or CS and which were associated with point mutations and deletions. For details of TFBSs see Supplementary Table S1.

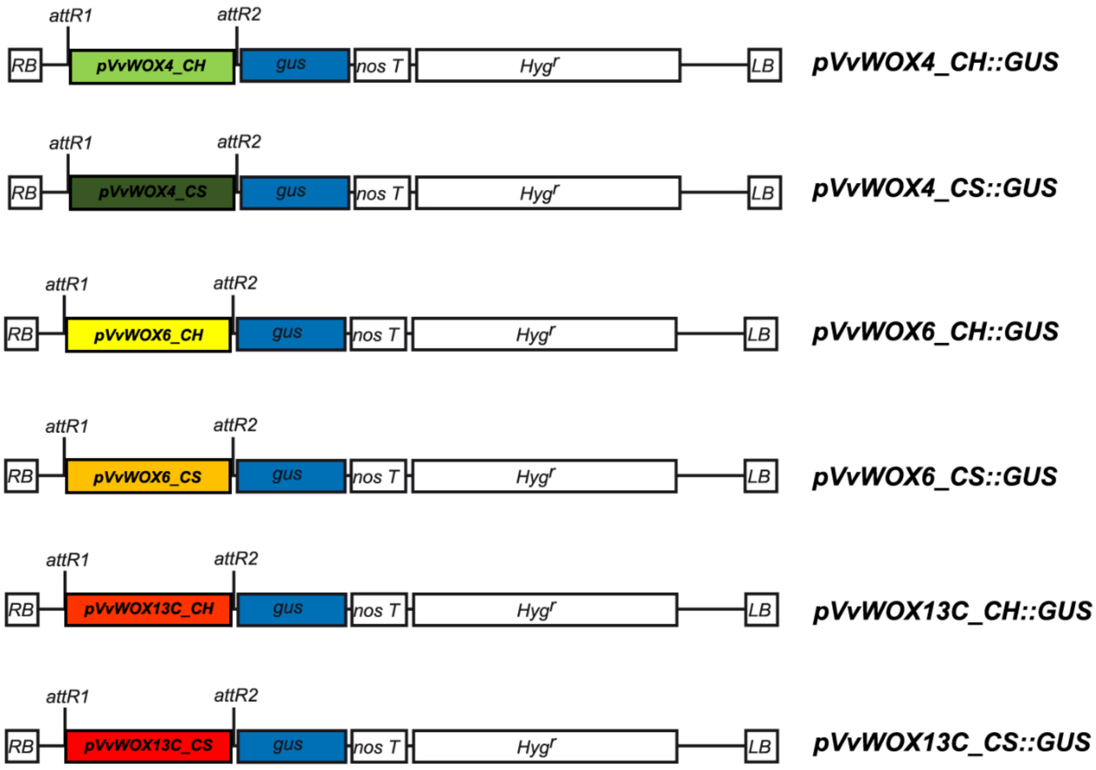

Supplementary Figure S3. Schematic representation of promoter::*GUS* fusion constructs inserted in expression vector pMDC164. RB: right border; attR1 and attR2: recombination sites for Gateway® Technology; *GUS*: β-glucunoridase gene; nosT: nos terminator; Hyg^r: Hygromycin resistance gene; LB: left border. *pVvWOX4*_CH: promoter of *VvWOX4* isolated from 'Chardonnay' (CH). *pVvWOX4*_CS: promoter of *VvWOX4* isolated from 'Cabernet Sauvignon' (CS). *pVvWOX6*_CH: promoter of *VvWOX6* isolated from 'Chardonnay' (CH). *pVvWOX6*_CS: promoter of *VvWOX6* isolated from 'Cabernet Sauvignon' (CS). *pVvWOX13C*_CH: promoter of *VvWOX13C* isolated from 'Chardonnay' (CH). *pVvWOX13C*_CS: promoter of *VvWOX13C* isolated from 'Cabernet Sauvignon' (CS).

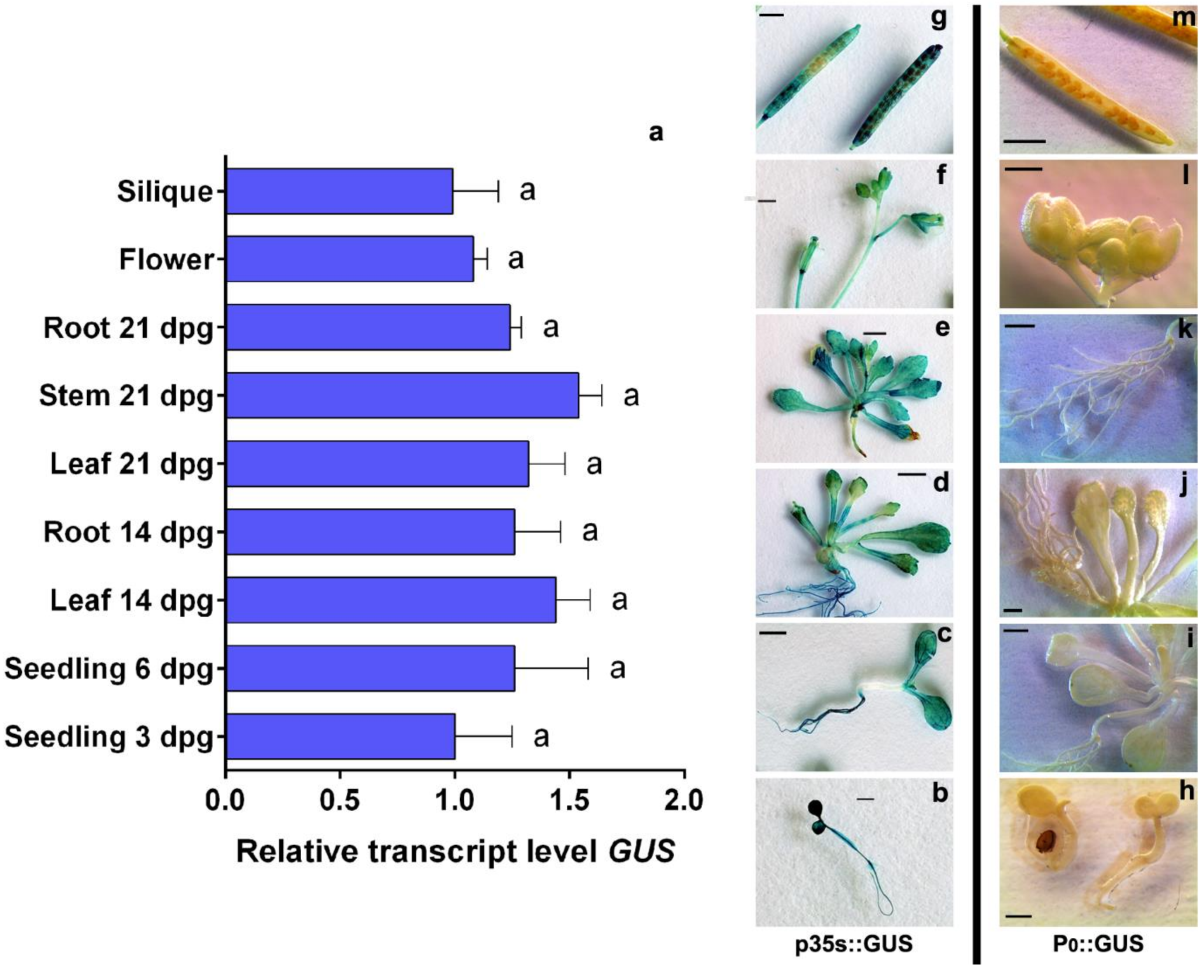

Supplementary Figure S4. Characterisation of *p35S::GUS* and empty vector without promoter (*p0::GUS*) in *Arabidopsis thaliana*. a) Relative expression level of *GUS* under control of p35S in transgenic *Arabidopsis* organs collected in different developmental stages. Lowercase letters denote significant differences attested by Tukey's HSD test ($P < 0.05$). Data are expressed as means ± SE (n = 3). b-m) Histochemical assay for GUS expression in transgenic *Arabidopsis* organs. Data shown are representative of three independent transgenic lines. Size bar = 2 mm.

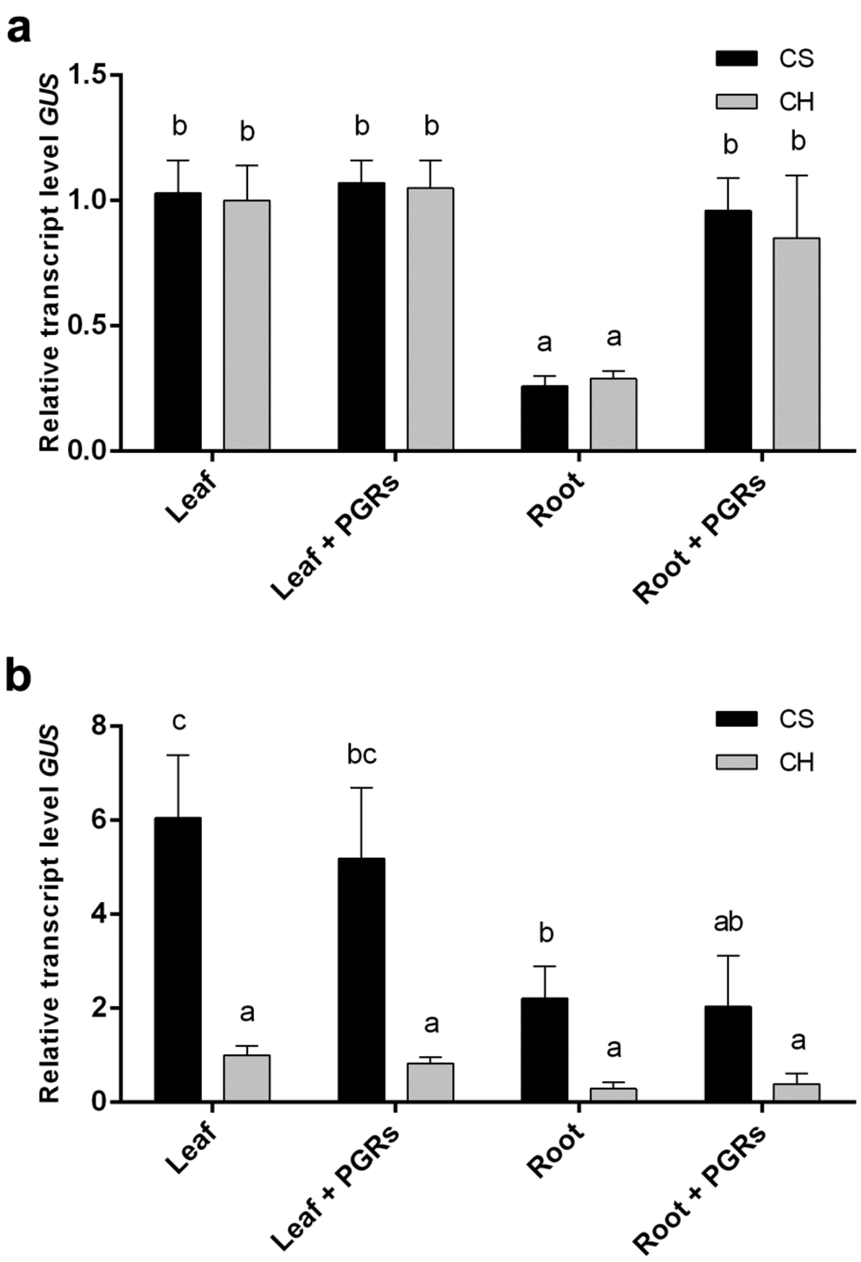

Supplementary Figure S5. Relative expression level of *GUS* under control of promoters: a) *pVvWOX4_CH* and *pVvWOX4_CS*; b) *pVvWOX13C_CH* and *pVvWOX13C_CS* in transgenic *Arabidopsis*. The organs were collected in standard culture conditions (leaf and root) and after 8 days of culture with the plant growth regulators 2,4 D and BA (leaf + PGR and root + PGR). Lowercase letters denote significant differences attested by Tukey's HSD test ($P < 0.05$). Data are expressed as means ± SE (n = 3).

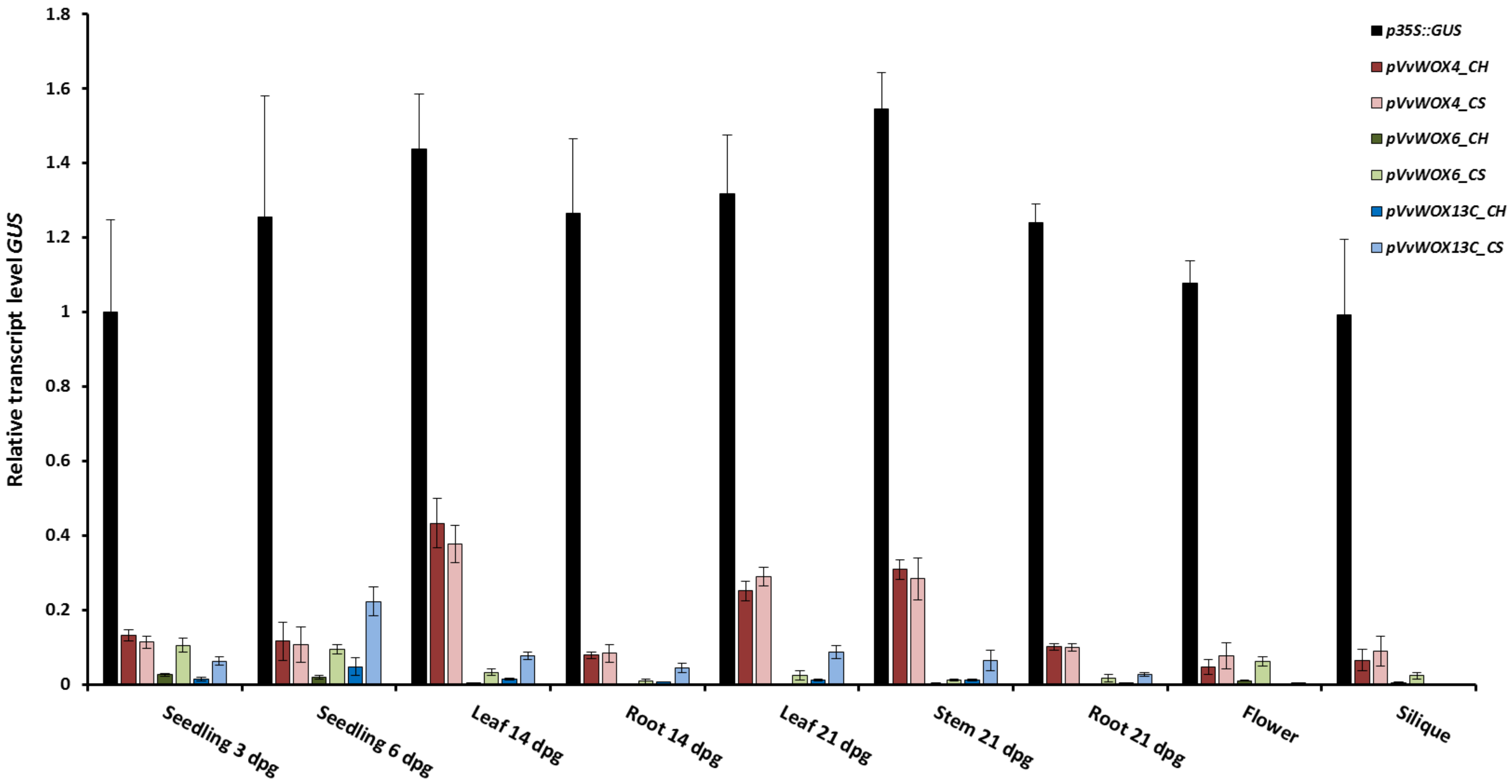

Supplementary Figure S6. Relative expression level of *GUS* under control of promoters: *p35S*, *pVvWOX4_CH*, *pVvWOX4_CS*, *pVvWOX6_CH*, *pVvWOX6_CS*, *pVvWOX13C_CH* and *pVvWOX13C_CS* in transgenic *Arabidopsis*. All data refer to seedling at 3 dpg transformed with *p35S::GUS*. Data are expressed as means ± SE (n = 3).
